# MEX3B inhibits collagen production in eosinophilic nasal polyps by downregulating epithelial cell *TGFBR3* mRNA stability

**DOI:** 10.1172/jci.insight.159058

**Published:** 2023-05-08

**Authors:** Jin-Xin Liu, Ao-Nan Chen, Qihong Yu, Ke-Tai Shi, Yi-Bo Liu, Cui-Lian Guo, Zhe-Zheng Wang, Yin Yao, Li Pan, Xiang Lu, Kai Xu, Heng Wang, Ming Zeng, Chaohong Liu, Robert P. Schleimer, Ning Wu, Bo Liao, Zheng Liu

**Affiliations:** 1 Department of Otolaryngology-Head and Neck Surgery, Tongji Hospital; and; 2 Hepatic Surgery Center, Tongji Hospital, Tongji Medical College, Huazhong University of Science and Technology, Wuhan, China.; 3 Clinical Medical Research Center of Hepatic Surgery at Hubei Province, Wuhan, China.; 4 Department of Pathogen Biology, School of Basic Medicine, Tongji Medical College, Huazhong University of Science and Technology, Wuhan, China.; 5 Division of Allergy-Immunology, Department of Medicine; and; 6 Department of Otolaryngology, Northwestern University Feinberg School of Medicine, Chicago, Illinois, USA.; 7 Department of Immunology, School of Basic Medicine, Tongji Medical College, and; 8 Cell Architecture Research Center, Huazhong University of Science and Technology, Wuhan, China.

**Keywords:** Inflammation, Allergy, Collagens

## Abstract

Although the expression of Mex3 RNA-binding family member B (MEX3B) is upregulated in human nasal epithelial cells (HNECs) predominately in the eosinophilic chronic rhinosinusitis (CRS) with nasal polyps (CRSwNP) subtype, its functions as an RNA binding protein in airway epithelial cells remain unknown. Here, we revealed the role of MEX3B based on different subtypes of CRS and demonstrated that MEX3B decreased the TGF-β receptor III (*TGFBR3*) mRNA level by binding to its 3′ UTR and reducing its stability in HNECs. TGF-βR3 was found to be a TGF-β2–specific coreceptor in HNECs. Knocking down or overexpressing MEX3B promoted or inhibited TGF-β2–induced phosphorylation of SMAD2 in HNECs, respectively. TGF-βR3 and phosphorylated SMAD2 levels were downregulated in CRSwNP compared with controls and CRS without nasal polyps with a more prominent downregulation in the eosinophilic CRSwNP. TGF-β2 promoted collagen production in HNECs. Collagen abundance decreased and edema scores increased in CRSwNP compared with control, again more prominently in the eosinophilic type. Collagen expression in eosinophilic CRSwNP was negatively correlated with MEX3B but positively correlated with TGF-βR3. These results suggest that MEX3B inhibits tissue fibrosis in eosinophilic CRSwNP by downregulating epithelial cell *TGFBR3* expression; consequently, MEX3B might be a valuable therapeutic target against eosinophilic CRSwNP.

## Graphical abstract

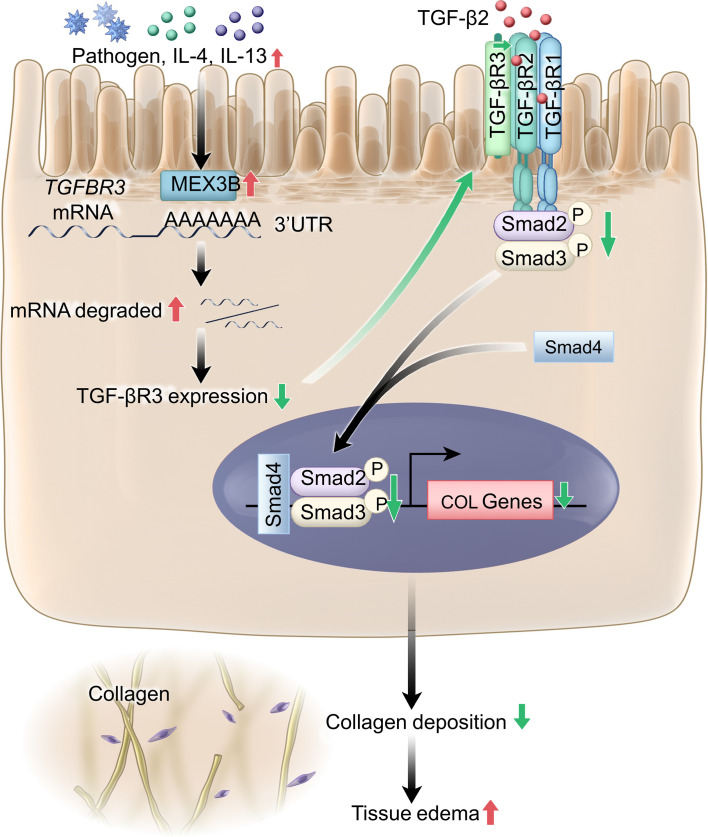

## Introduction

The respiratory epithelium provides a physical, functional, and immunologic barrier to protect the host from the potential harmful effects of inhaled environmental infectious pathogens, pollutants, and allergens and to guarantee the maintenance of a healthy host state (1, 2). Epithelial cell dysfunction is central to the pathogenesis of airway diseases including asthma, chronic obstructive pulmonary disease, allergic rhinitis, and chronic rhinosinusitis (CRS) (1–4). Recently, we have discovered that the expression of Mex3 RNA-binding family member B (MEX3B) was significantly elevated in nasal epithelial cells in CRS (5). MEX3B has been found to function as a coreceptor of TLR3 and promote TLR3 ligand-induced thymic stromal lymphopoietin production in nasal epithelial cells (5), thus contributing to the development of type 2 and eosinophilic inflammation in CRS. Nevertheless, MEX3B primarily is an RNA-binding protein (RBP) that contains 2 K homology-type (KH-type) RNA recognition domains (6, 7). RBPs are central effectors in the control of posttranscriptional mechanisms that contribute to multiple steps of RNA metabolism including pre-mRNA splicing, mRNA turnover, mRNA stability, and mRNA translation (6–11). Previous studies indicate diverse roles of MEX3B in regulating RNA metabolism in a cell-dependent and context-specific manner. MEX3B has been demonstrated to impair *HLA-A* mRNA stability by binding to its 3′ UTR in melanoma cells (12). In contrast, MEX3B might stabilize *CXCL2* mRNA and upregulated *CXCL2* expression in the murine respiratory epithelial cell–derived cell line MLE-15 (13). Nevertheless, the roles of MEX3B as an RBP in human diseases remain largely unknown.

CRS is a disturbing respiratory inflammatory disorder of the mucosa of the nose and paranasal sinuses, affecting about 10% of the population globally (14–17). According to the presence or absence of nasal polyps (NPs), CRS is classified into chronic rhinosinusitis with nasal polyps (CRSwNP) and chronic rhinosinusitis without nasal polyps (CRSsNP) (14–16, 18). CRSwNP can be further divided into eosinophilic and noneosinophilic types based on the extent of tissue eosinophil infiltration (14, 18, 19). These CRS subtypes demonstrate considerable differences in inflammatory and tissue remodeling features, associated with distinct responses to medical and surgical treatments (19–22). Generally, eosinophilic CRSwNP is characterized by a predominant type 2 response and significant edema formation, whereas noneosinophilic CRSwNP demonstrates a skewed type 1/type 17 response and less edema formation (17, 18, 22, 23). CRSsNP also has less eosinophil infiltration but is associated with significant tissue fibrosis, accompanied by elevated levels of TGF-β1 signaling activation (18, 22, 24). Although the tissue remodeling patterns of CRS subtypes have been established (22, 25), the contribution and regulation of different TGF-β isoforms and their corresponding receptors in the tissue remodeling in CRS are still undetermined (22, 26, 27).

In this study, by using the combination of improved RNA IP-coupled high-throughput sequencing (iRIP-Seq), gene knockdown and overexpression, RNA-Seq, and histology of nasal mucosa tissue and NP tissue, we performed a detailed identification of MEX3B targets in nasal epithelial cells and explored their contribution to CRS pathogenesis. Establishing a better mechanistic understanding of MEX3B in human diseases may help develop potentially novel approaches for targeting disease-specific pathobiology.

## Results

### The expression and regulation of MEX3B in nasal epithelial cells.

**Figure 1:**
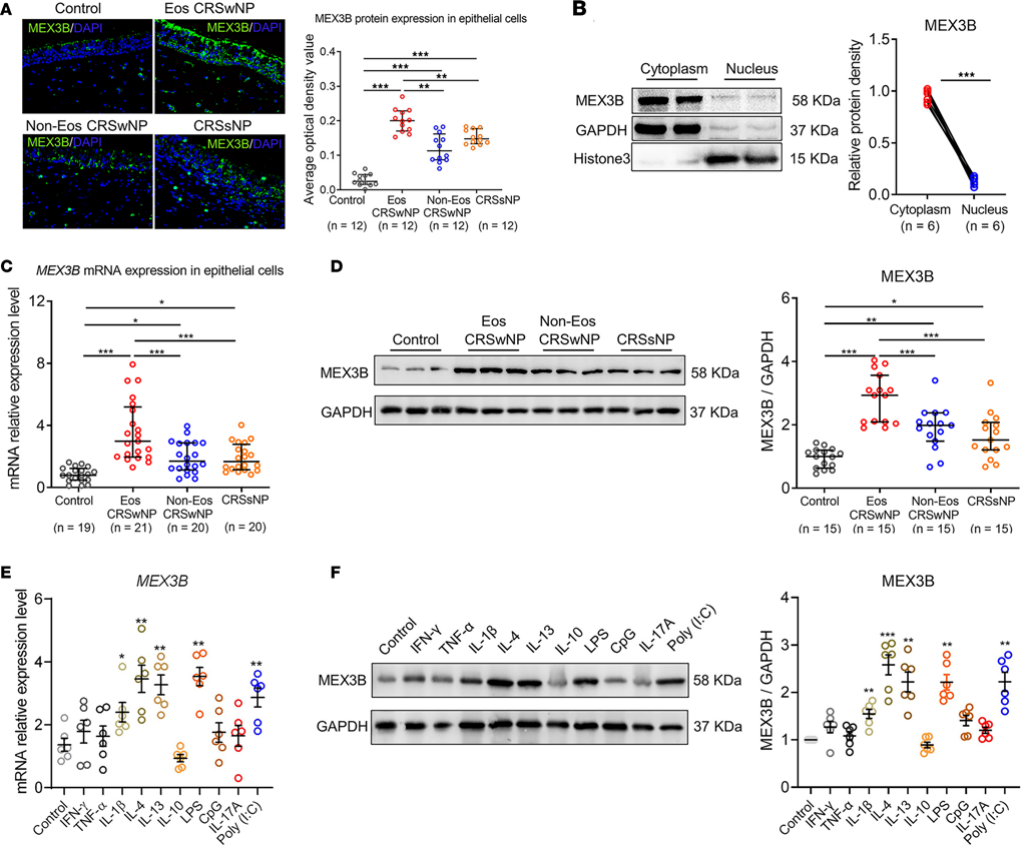
MEX3B mRNA and protein expression are upregulated in epithelial cells in CRS. (**A**) Immunoreactivity of MEX3B in nasal epithelial cells in different study groups as detected by immunofluorescence staining. The representative photomicrographs are shown. Original magnification ×400. (**B**) MEX3B levels in cytoplasm and nucleus in nasal epithelial cells obtained from patients with eosinophilic CRSwNP (*n* = 6). Representative Western blotting images are shown, and densitometric analysis of blots was performed. Data are presented in dot plots and were analyzed by paired Student’s *t* test. Symbols represent individual samples. (**C**) *MEX3B* mRNA expression in nasal epithelial cells in different study groups as detected by quantitative RT-PCR. (**D**) The protein levels of MEX3B in nasal epithelial cells in different study groups as determined by Western blotting. Representative Western blotting images are shown and densitometric analysis of blots was performed. (**E** and **F**) HNECs obtained from controls were cultured with the ALI method and stimulated with various cytokines and TLR agonists (*n* = 6). After 6-hour stimulation, cells were subjected to quantitative RT-PCR analysis of *MEX3B* mRNA expression (**E**). After 24-hour stimulation, cells were subjected to Western blotting analysis of MEX3B protein expression (**F**). Representative blots are shown and densitometric analysis of blots was performed. For **A**, **C**, and **D**, data are presented as median and interquartile range and were analyzed by Kruskal-Wallis test with Dunn’s post hoc test. For **E** and **F**, data are presented as the mean ± SEM and were analyzed by unpaired Student’s *t* test. *P < 0.05, **P < 0.01, and ***P < 0.001. Eos, eosinophilic; Non-Eos, non-eosinophilic.

As an extension of our previous study (5), we examined the tissue-specific cellular expression of MEX3B in sinonasal mucosa. We found that MEX3B was mainly expressed by nasal epithelial cells, and its expression was upregulated in patients with CRS compared with control participants by immunofluorescence staining (Figure 1A). In addition, infiltrating cells including c-kit^+^ mast cells, CD68^+^ macrophages, myeloperoxidase^+^ neutrophils, and CD20^+^ B cells also had MEX3B expression in mucosa from patients with CRS (Supplemental Figure 1; supplemental material available online with this article; https://doi.org/10.1172/jci.insight.159058DS1). A blocking experiment with recombinant human MEX3B protein was performed to demonstrate the specificity of used polyclonal anti-MEX3B Ab (Supplemental Figure 2). Given the importance of epithelial cells in regulating the airway pathophysiological process, we next concentrated on the function of MEX3B in nasal epithelial cells. RBPs may possess different functions depending on their intracellular localization. Immunofluorescence staining demonstrated predominant localization of MEX3B immunoreactivity in cytoplasm of nasal epithelial cells (Figure 1A). Further Western blotting analysis consistently revealed a significant presence of MEX3B in cytoplasm rather than in the nucleus in epithelial cells from patients with eosinophilic CRSwNP (Figure 1B). These results suggest a possible role of MEX3B in controlling mRNA stability or translation as an RBP.

In this study, we verified the upregulation of mRNA and protein expression of MEX3B in nasal epithelial cells from patients with CRS as compared with epithelial cells from control participants without CRS, with a more profound increase in eosinophilic CRSwNP than in noneosinophilic CRSwNP and CRSsNP (Figure 1, C and D). Importantly, we found that MEX3B mRNA and protein levels were upregulated by IL-1β, IL-4, IL-13, polyinosinic: polycytidylic acid (poly [I:C]), and LPS in air-liquid interface–cultured (ALI-cultured) human nasal epithelial cells (HNECs) obtained from controls, while no significant effect was observed for IFN-γ, TNF-α, IL-10, CpG, or IL-17A (Figure 1, E and F).

### MEX3B downregulates TGF-β receptor III gene expression in nasal epithelial cells.

**Figure 2:**
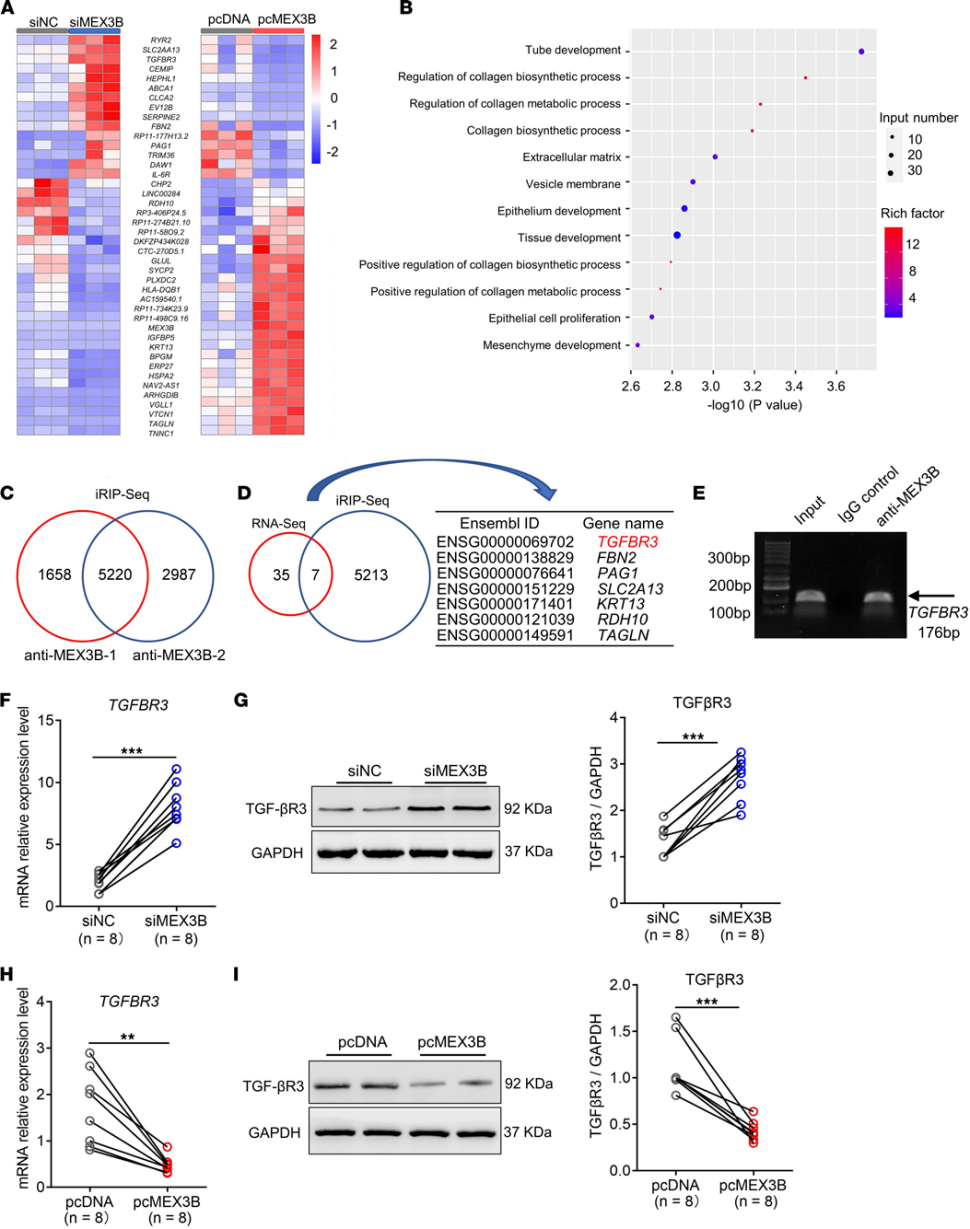
*TGFBR3* mRNA is the direct target of MEX3B in nasal epithelial cells. (**A**) The heat map shows genes regulated by MEX3B in BEAS-2B cells as detected by RNA-Seq. Forty-two genes altered in a consistent way upon siMEX3B and pcMEX3B treatment are shown (*n* = 3 for each group). (**B**) Dot bubble graph shows the GO functional enrichment analysis for the 42 genes shown in **A**. (**C**) Venn diagrams of overlapping genes targeted by MEX3B in BEAS-2B cells in 2 iRIP-Seq assays. (**D**) Venn diagrams of overlapping genes directly bound and regulated by MEX3B in BEAS-2B cells according to RNA-Seq and iRIP-Seq assay. (**E**) Confirmation of *TGFBR3* mRNA as a MEX3B binding RNA in HNECs by RIP assay followed by RT-PCR (*n* = 6). Representative agarose gel electrophoresis results are shown. After siMEX3B transfection, *TGFBR3* mRNA and protein expression in ALI-cultured HNECs obtained from controls were detected by RT-PCR (**F**) and Western blotting (**G**) (*n* = 8), respectively. Representative blots are shown and densitometric analysis was performed. After pcMEX3B transfection, *TGFBR3* mRNA and protein expression in ALI-cultured HNECs was detected by RT-PCR (**H**) and Western blotting (**I**), respectively (*n* = 8). Representative blots are shown and densitometric analysis was performed. For **F**–**I**, data are presented in dot plots and were analyzed by paired Student’s *t* test. Symbols represent individual samples. **P < 0.01 and ***P < 0.001.

To dissect the function of MEX3B in nasal epithelial cells, we first investigated the overall profile of mRNAs regulated by MEX3B in BEAS-2B cells, a human bronchial epithelial cell line. We identified 42 genes that were consistently altered following *MEX3B* knockdown (small interfering MEX3B [siMEX3B] treatment) and overexpression (plasmid containing MEX3B [pcMEX3B] treatment) in BEAS-2B cells (Figure 2A). Gene ontology (GO) analysis indicated that these genes are mainly involved in collagen biosynthetic processes, extracellular matrix (ECM) function, and epithelium and tissue development (Figure 2B). Next, iRIP-Seq was carried out to identify transcriptome-wide target RNAs of MEX3B as an RBP in BEAS-2B cells, and 5,220 gene transcripts were consistently identified by 2 independent experiments (Figure 2C). The overlapping mRNAs in RNA-Seq and iRIP-Seq experiments are considered as those bound and regulated by MEX3B in BEAS-2B cells, and we identified 7 overlapping genes (Figure 2D). Among them, TGF-β receptor III gene (*TGFBR3*) is of particular interest. *TGFBR3* encodes TGF-βR3, a specific TGF-β2 coreceptor and critical regulator of collagen production (28, 29). TGF-β signaling is activated by the binding of the TGF-β ligand to cell surface receptors, and 3 isoforms (TGF-β 1, 2, and 3) display distinct affinities for extracellular ligand binding domains of different subfamilies of TGF-β receptors (TGF-βR1, 2, and 3) (25, 28–30). TGF-βR3 promotes TGF-β2 signaling by forming the TGF-β2/TGF-βR3 complex and then recruiting and activating TGF-βR2 and TGF-βR1 (28, 29, 31).

To verify the BEAS-2B cell findings in HNECs, we performed the RNA IP assay using HNECs obtained from controls. By subsequent reverse transcription PCR (RT-PCR) detection, we verified that the co-IP of *TGFBR3* mRNA with MEX3B was substantially enriched compared with the level of co-IP using control rabbit IgG (Figure 2E), suggesting that MEX3B directly associates with *TGFBR3* mRNA. We also verified that TGF-βR3 expression was upregulated by siMEX3B treatment and downregulated by pcMEX3B treatment in ALI-cultured HNECs at both the mRNA and protein levels (Figure 2, F–I). In contrast, we failed to discover any obvious effect of MEX3B on expression of *TGFBR1* or *TGFBR2* in ALI-cultured HNECs (Supplemental Figure 3). We overexpressed β-glucuronidase (*GUSB*) encoding β-glucuronidase as a non-MEX protein control, to eliminate the influence of stress upon forcing cells to overexpress a protein under the CMV promotor of pcDNA. We found that pcGUSB had no effect on TGF-βR3 expression in ALI-cultured HNECs at both mRNA and protein levels (Supplemental Figure 4). Transfection efficiencies of MEX3B in ALI-cultured HNECs and BEAS-2B cells were verified at both mRNA and protein levels (Supplemental Figure 5).

### MEX3B reduces TGFBR3 mRNA stability via binding to its 3′ UTR.

**Figure 3:**
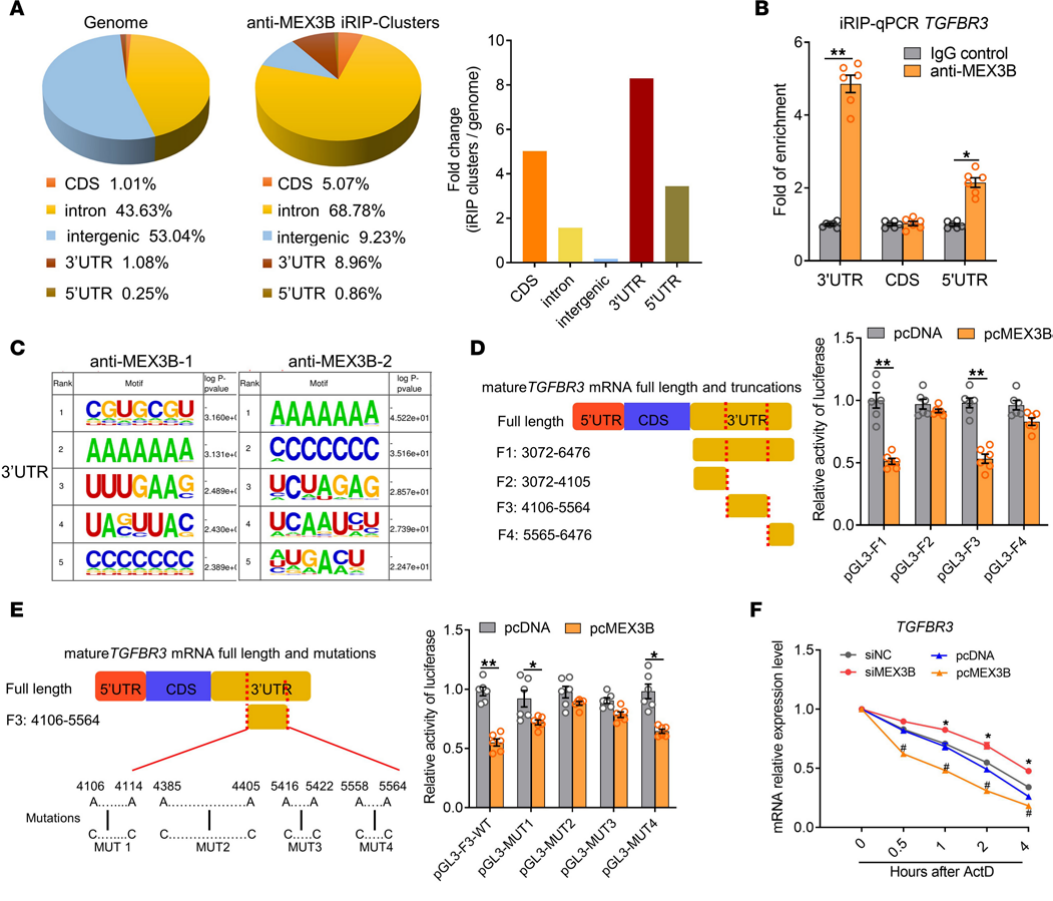
MEX3B binds to 3′ UTR of TGFBR3 mRNA and destabilizes it. (**A**) Distribution of MEX3B binding sites among coding exons (CDS), introns, intergenic, 5′ UTRs, and 3′ UTRs were calculated (right) based on 2 iRIP-Seq assays in BEAS-2B cells. The fold-changes of iRIP cluster distribution relative to genomic distribution were calculated. (**B**) The enrichment of MEX3B binding sites in TGFBR3 mRNA in HNECs studied by RIP assay followed by RT-PCR (n = 6). Data are presented as the mean ± SEM and were analyzed by the Mann-Whitney U 2-tailed test. (**C**) Sequences of MEX3B binding cluster motifs in 3′ UTRs were analyzed by HOMER to identify enriched binding motifs. The top 5 most-enriched motifs and their P values in the 2 iRIP-Seq assays are shown. (**D**) The DNA fragments from the human TGFBR3 3′ UTR were amplified and subcloned into a firefly luciferase reporter pGL3. HNECs were cotransfected with the indicated pGL3-TGFBR3 and pcMEX3B. Cell lysates were harvested for the luciferase assay (n = 6). (**E**) A schematic presentation of potential MEX3B binding sites in 3′ UTR TGFBR3 mRNA and the corresponding mutants are shown. HNECs were cotransfected with the indicated mutated pGL3-TGFBR3 and pcMEX3B. Cell lysates were harvested for the luciferase assay (n = 6). For D and E, data are presented as the mean ± SEM and were analyzed by unpaired Student’s t test. (**F**) TGFBR3 mRNA stability detected by means of RT-PCR in siMEX3B or pcMEX3B transfected ALI-cultured HNECs after treatment with actinomycin D (ActD) at 5 μg/mL (n = 6). Data are presented as the mean and were analyzed by the paired Student’s t test. *P < 0.05, **P < 0.01, and #P < 0.05 compared with their corresponding control group.

To gain mechanistic insights into MEX3B function, we utilized iRIP-Seq results to map the locations of transcriptome-wide MEX3B binding sites in BEAS-2B cells. We found that the canonical binding sites of MEX3B were located within introns (68.78%), 3′ UTRs (8.96%), coding sequences (5.07%), and 5′ UTRs (0.86%) (Figure 3A). Notably, MEX3B binding sites were most highly enriched in the 3′ UTRs compared with other structures relative to their genomic sizes, indicating the possibility of preferential binding to the 3′ UTRs in BEAS-2B cells (Figure 3A). The iRIP clusters represent predicted binding sites in all potential target RNAs. To explore the specific MEX3B binding sites on *TGFBR3* mRNA in HNECs, we designed specific primers for different structures of mature *TGFBR3* mRNA and performed PCR assay after RNA IP with anti-MEX3B Ab in HNECs. We found that MEX3B preferentially bound to the 3′ UTR of *TGFBR3* mRNA in HNECs (Figure 3B).

Sequences of *TGFBR3* mRNA binding clusters in 3′ UTR were analyzed by HOMER to identify enriched binding motifs, and it was revealed that MEX3B binding sites were most likely enriched in AAAAAAA motifs in the 3′ UTR (Figure 3C). The AAAAAAA motif is enriched in the 4106 to 5564 (F3) segment relative to the transcriptional start site (TSS) in the 3′ UTR of *TGFBR3* mRNA (Supplemental Figure 6). Truncated 3′ UTR of *TGFBR3* mRNA was generated by PCR and subcloned into firefly luciferase reporter construct pGL3 (Figure 3D). To confirm the binding sites on mature *TGFBR3* mRNA, submerged cultured HNECs were cotransfected with pcMEX3B and luciferase reporter constructs. Dual luciferase assay results showed that depletion of the 4106 to 5564 (F3) segment in the 3′ UTR abolished the inhibitory effect of MEX3B on the luciferase activity (Figure 3D). In the 4106 to 5564 (F3) segment of the 3′ UTR of *TGFBR3* mRNA*,* there are 4 AAAAAAA-enriched putative MEX3B binding sites (Supplemental Figure 6). We therefore prepared constructs with A-to-C mutations in these 4 sites, including 4106 to 4114 sites (MUT1), 4385 to 4405 sites (MUT2), 5416 to 5422 sites (MUT3), and 5558 to 5564 sites (MUT4) (Figure 3E). MUT2 and MUT3 diminished the inhibitory effect of MEX3B on expression of luciferase activity in submerged cultured HNECs (Figure 3E). Last, we explored whether the MEX3B affects *TGFBR3* mRNA stability. We found that the stability of *TGFBR3* mRNA was enhanced after siMEX3B treatment and decreased after pcMEX3B treatment, compared with the corresponding control in ALI-cultured HNECs (Figure 3F). Taken together, these data indicate that MEX3B can bind to the AAAAAAA motifs located in the 4385 to 4405 sites and the 5416 to 5422 sites in the 3′ UTR of *TGFBR3* mRNA and destabilize the mRNA, thus downregulating TGF-βR3 expression.

### TGF-βR3 expression is downregulated in nasal epithelial cells in CRSwNP.

**Figure 4:**
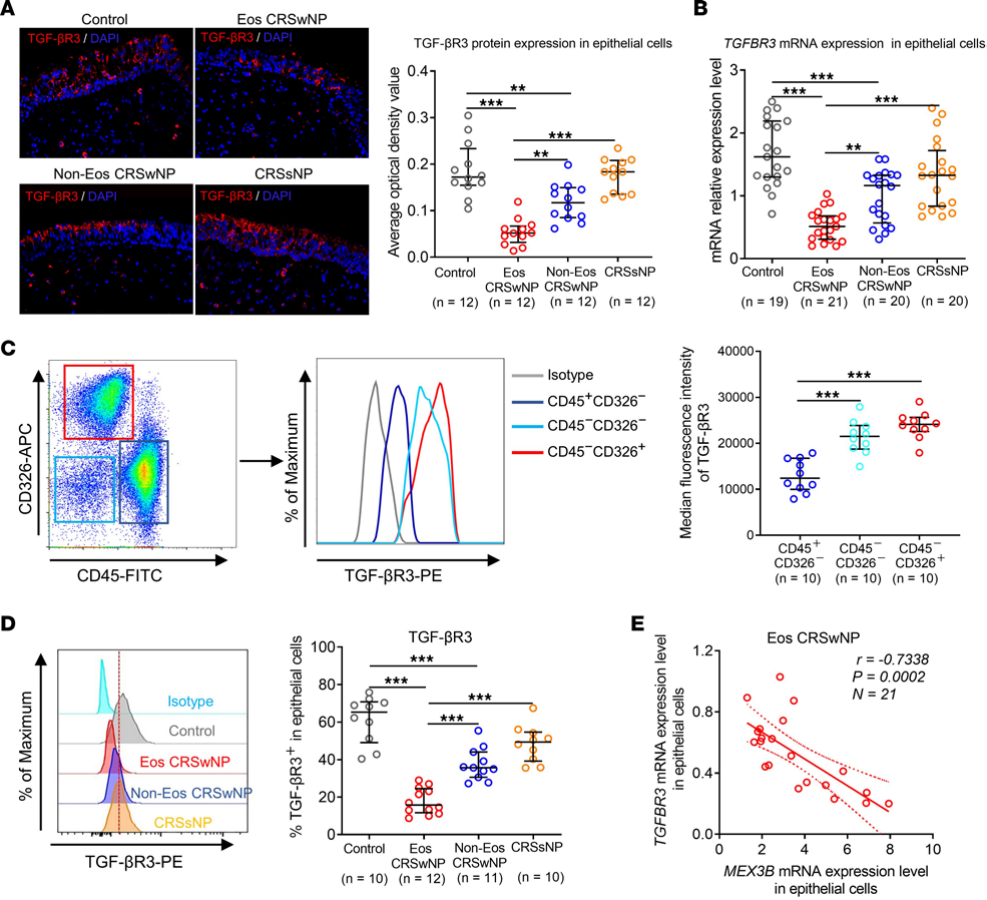
Expression of *TGFBR3* mRNA and protein is downregulated in epithelial cells in eosinophilic CRSwNP. (**A**) Immunoreactivity of TGF-βR3 in nasal epithelial cells in different study groups as detected by immunofluorescence staining. The representative photomicrographs are shown. Original magnification ×400. (**B**) *TGFBR3* mRNA expression in nasal epithelial cells in different study groups as detected by quantitative RT-PCR. (**C**) Dispersed nasal tissue cells from controls (*n* = 10) were subjected to flow cytometric analysis of cell surface expression of TGF-βR3 in CD45^+^CD326^–^ hematopoietic cells, CD45^–^CD326^–^ stromal cells, and CD45^–^CD326^+^ epithelial cells. A representative histogram is shown and the staining intensities are quantified. (**D**) Flow cytometric analysis of the frequencies of TGF-βR3–positive cells in CD45^–^CD326^+^ epithelial cells in different participant groups. Representative histograms are shown. For **A**–**D**, data are presented as median and interquartile range and were analyzed by the Kruskal-Wallis test with Dunn’s post hoc test. (**E**) The *MEX3B* mRNA expression levels negatively correlated with the *TGFBR3* mRNA expression levels in nasal epithelial cells in eosinophilic patients with CRSwNP. Spearman’s correlation was used for correlation analysis. **P < 0.01 and ***P < 0.001. Eos, eosinophilic; Non-Eos, non-eosinophilic.

Next, to determine whether these in vitro findings have relevance in vivo in patients, we explored the association between MEX3B and TGF-βR3 expression in tissue derived from patients with different CRS subtypes. Immunofluorescence staining revealed that TGF-βR3 was abundantly expressed by epithelial cells in controls and patients with CRSsNP (Figure 4A). The staining intensity of TGF-βR3 in epithelial cells was significantly lower in CRSwNP than in controls, with eosinophilic CRSwNP having the lowest expression (Figure 4A). In agreement with findings based on immunofluorescence staining, we observed a significant downregulation of epithelial *TGFBR3* mRNA expression in tissue from eosinophilic CRSwNP as compared with other types of CRS and controls (Figure 4B).

Some TGF-βR3^+^ cells were also observed in the lamina propria in sinonasal mucosa (Figure 4A). We then compared TGF-βR3 expression intensity in epithelial cells, stromal cells, and inflammatory cells in lamina propria by flow cytometric analysis of dispersed nasal tissue cells from controls using staining of CD326 (EpCAM), an epithelial marker. We found that CD45^+^CD326^–^ hematopoietic cells had lower TGF-βR3 expression than CD45^–^CD326^–^ stromal cells and CD45^–^CD326^+^ epithelial cells (Figure 4C). In addition, the abundance of stromal cells was much less than that of epithelial cells in nasal tissues (Figure 4C). We therefore retained our interest in epithelial cell TGF-βR3. By flow cytometric analysis, we consistently found that the frequencies of TGF-βR3^+^ epithelial cells were markedly reduced in eosinophilic CRSwNP compared with other types of CRS and controls (Figure 4D). Notably, there was a negative correlation between the levels of expression of mRNA for *MEX3B* and *TGFBR*3 in nasal epithelial cells in eosinophilic CRSwNP (Figure 4E).

### Reduced activation of TGF-β signaling in CRSwNP.

TGF-β family members are master switches in the induction of ECM (25, 30, 32). TGF-β1 and TGF-β3 bind to TGF-βR1 and TGF-βR2 directly, whereas TGF-β2 binds to TGF-βR3 initially and then recruits and activates TGF-βR2 and TGF-βR1 (28, 31, 33). To study the role of TGF-β isoforms and their receptors in CRS, we measured their expression in different CRS subtypes. We found that the levels of TGF-β1 mRNA and protein were decreased in NP tissues from patients with eosinophilic and noneosinophilic CRSwNP, whereas TGF-β1 was increased in diseased sinus mucosa tissues from patients with CRSsNP compared with controls (Supplemental Figure 7, A and D). The mRNA and protein levels of TGF-β2 were upregulated in all types of CRS compared with controls (Supplemental Figure 7, B and E), while no significant differences in levels of TGF-β3 mRNA and protein were found among different study groups (Supplemental Figure 7, C and F). In addition, we found an upregulation of mRNA expression of *TGFBR1* and *TGFBR2* in nasal epithelial cells from patients with CRSsNP, but no change was found in epithelial cells from patients with eosinophilic or noneosinophilic CRSwNP, compared with controls (Supplemental Figure 8).

**Figure 5:**
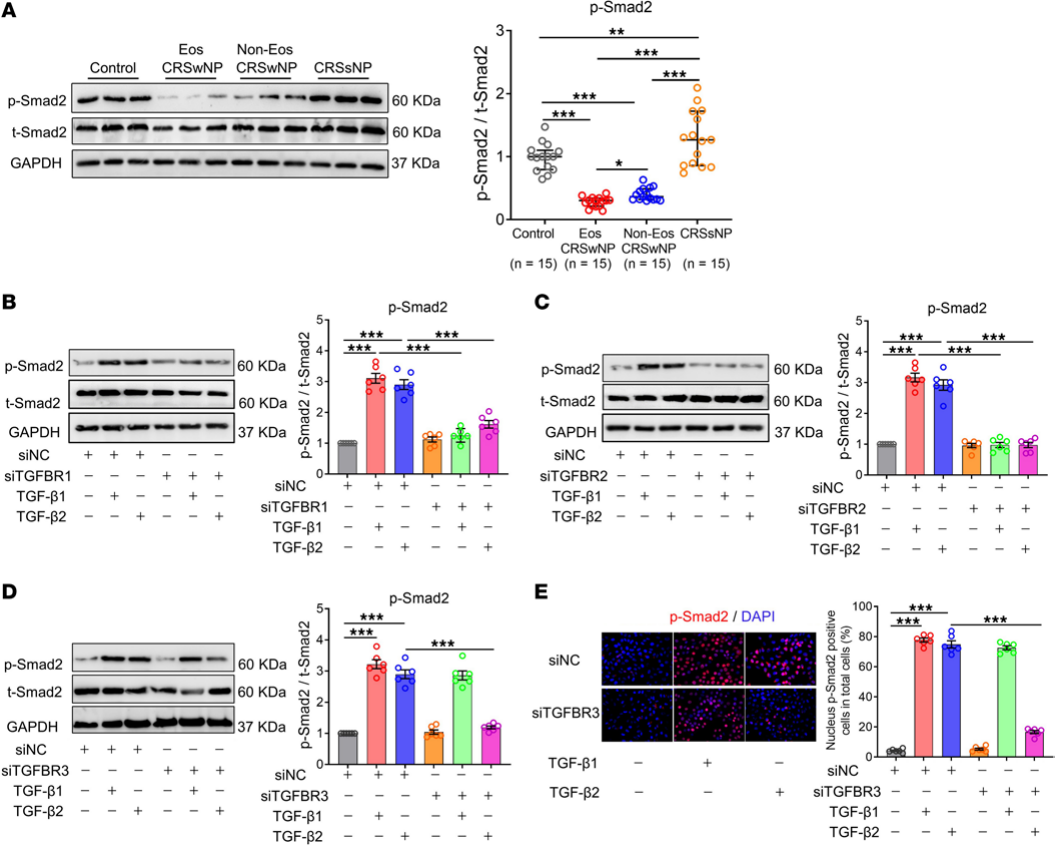
TGF-βR3 is indispensable for TGF-β2 signaling activation in nasal epithelial cells. (**A**) The protein levels of p-SMAD2 in sinonasal tissues from different study groups as measured by Western blotting. Representative blots are shown and densitometric analysis was performed. Data are presented as median and interquartile range, and were analyzed by the Kruskal-Wallis test with Dunn’s post hoc test. ALI-cultured HNECs obtained from controls were transfected with siTGFBR1 (**B**), siTGFBR2 (**C**), or siTGFBR3 (**D**), and then stimulated with TGF-β1 (10 ng/mL) or TGF-β2 (10 ng/mL). Thirty minutes after stimulation, the p-SMAD2 levels were detected by Western blotting (*n* = 6). Representative blots are shown and densitometric analysis was performed. (**E**) Submerged cultured HNECs were transfected with siTGFBR3 and stimulated with TGF-β1 (10 ng/mL) or TGF-β2 (10 ng/mL). Thirty minutes after stimulation, the nucleus p-SMAD2^+^ cells were detected by immunofluorescence staining (*n* = 6). The representative photomicrographs are shown. Original magnification ×400. For **B**–**E**, data are presented as the mean ± SEM and were analyzed by 1-way ANOVA with Tukey’s post hoc test. *P < 0.05, **P < 0.01, and ***P < 0.001. Eos, eosinophilic; Non-Eos, non-eosinophilic.

After activation of TGF-βR, phosphorylated SMAD2 and SMAD3 form heterodimers with SMAD4 and translocate to the nucleus (25, 30, 32). Here, we found decreased phosphorylated SMAD2 (p-SMAD2) levels in NP tissues from patients with CRSwNP, especially in the patients with eosinophilic type, but increased p-SMAD2 levels in diseased sinus mucosa tissues from CRSsNP compared with nasal tissues from controls (Figure 5A), suggesting an overall reduced TGF-β signaling in CRSwNP, but an elevated one in CRSsNP. The p-SMAD2 levels are consistent with TGF-β1 and TGF-βR3 expression patterns in different CRS subtypes, highlighting the importance of TGF-β1 and TGF-βR3 rather than other TGF-β isoforms or receptors in the pathogenesis of CRS.

Although TGF-βR3 function has been studied in myoblasts (28, 29), its function in airway epithelial cells has yet to be determined. Here, we found that siTGFBR1 and siTGFBR2 treatment abolished both TGF-β1– and TGF-β2–induced SMAD2 phosphorylation in ALI-cultured HNECs as detected by Western blotting (Figure 5, B and C) as well as flow cytometric analysis (Supplemental Figure 9, A and B). However, siTGFBR3 treatment only abolished TGF-β2–induced, but not TGF-β1–induced, SMAD2 phosphorylation in ALI-cultured HNECs (Figure 5D and Supplemental Figure 9C). This finding was further confirmed by immunofluorescence staining study showing reduced numbers of cells with p-SMAD2^+^ nuclei in siTGFBR3 transfected and submerged cultured HNECs after stimulation with TGF-β2, but not TGF-β1 (Figure 5E). Overall, these data indicate that TGF-βR3 is a TGF-β2–specific coreceptor and indispensable for TGF-β2 signaling in nasal epithelial cells. Furthermore, the loss of TGF-βR3 is likely to be involved in reduced TGF-β signaling activation in CRSwNP, particularly the eosinophilic type.

### MEX3B suppresses TGF-β2 signaling activation in nasal epithelial cells.

**Figure 6:**
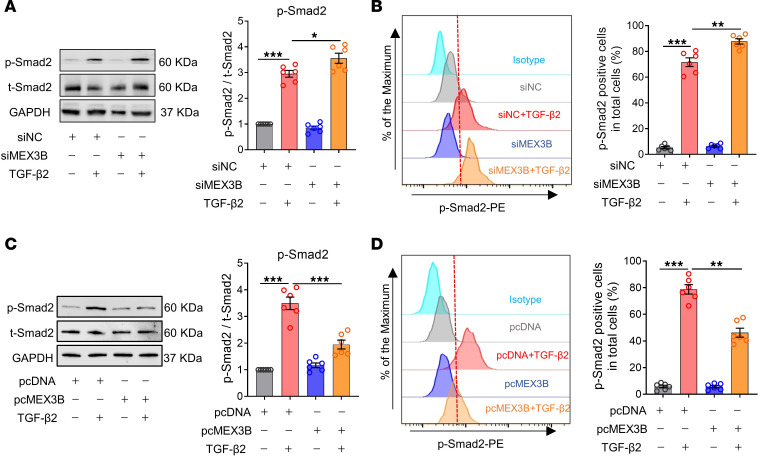
MEX3B suppresses TGF-β2 signaling activation in nasal epithelial cells. ALI-cultured HNECs obtained from controls were transfected with siMEX3B and subsequently stimulated with TGF-β2 (10 ng/mL). After 30-minute stimulation, the p-SMAD2 levels were detected by Western blotting (**A**) and flow cytometric analysis (**B**), respectively (*n* = 6). ALI-cultured HNECs were transfected with pcMEX3B and subsequently stimulated with TGF-β2 (10 ng/mL). After 30-minute stimulation, the p-SMAD2 levels were detected by Western blotting (**C**) and flow cytometric analysis (**D**), respectively (*n* = 6). Representative histograms are shown. For **A**–**D**, data are presented as the mean ± SEM and were analyzed by 1-way ANOVA with Tukey’s post hoc test. *P < 0.05, **P < 0.01, and ***P < 0.001.

Given the inhibitory effect of MEX3B on *TGFBR3* mRNA expression, we hypothesized that MEX3B is able to suppress TGF-β2 signaling activation in HNECs. Indeed, we found that MEX3B knockdown with siMEX3B significantly increased TGF-β2–induced SMAD2 phosphorylation in ALI-cultured HNECs (Figure 6, A and B). In contrast, overexpression of MEX3B with pcMEX3B transfection suppressed SMAD2 phosphorylation upon TGF-β2 treatment in ALI-cultured HNECs (Figure 6, C and D). Furthermore, we found that pcGUSB had no impact on the TGF-β2–induced SMAD2 phosphorylation in ALI-cultured HNECs (Supplemental Figure 10A). We also found that pcMEX3B had no effect on the IL-13–induced Stat6 phosphorylation in ALI-cultured HNECs (Supplemental Figure 10B). These findings confirm the specific effect of MEX3B on TGF-β2/TGF-βR3 signaling activation in HNECs.

### Loss of TGFBR3 expression inhibits TGF-β2–induced gene transcription in nasal epithelial cells.

**Figure 7:**
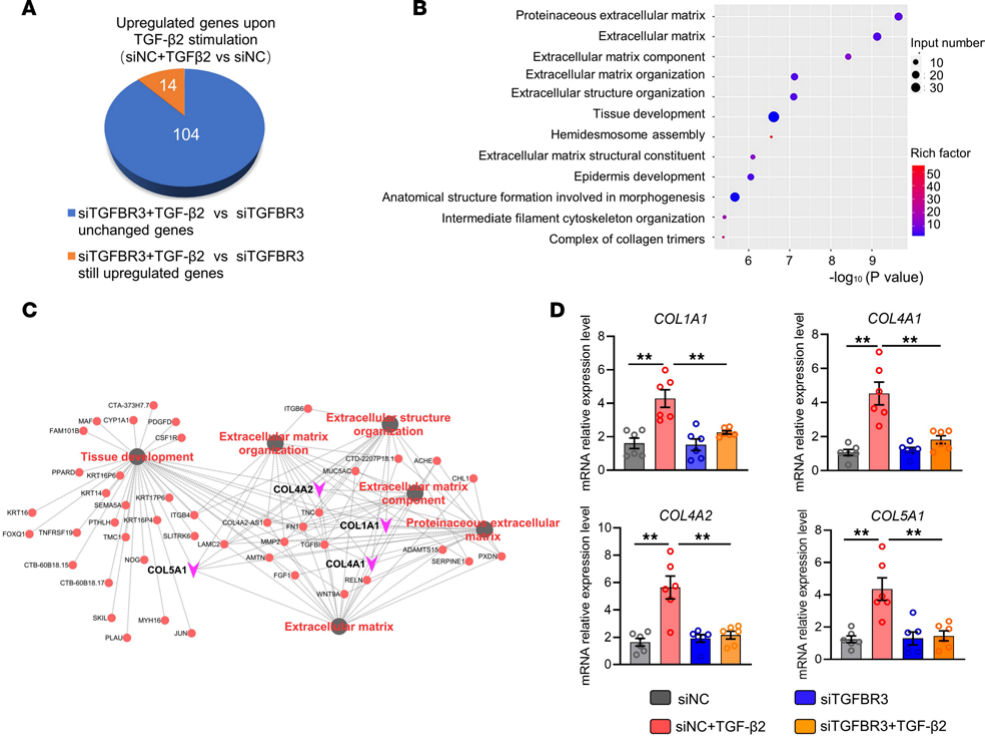
Loss of TGF-βR3 inhibits TGF-β2–induced collagen production in nasal epithelial cells. (**A**) Submerged cultured HNECs obtained from controls were transfected with siTGFBR3 or siNC and stimulated with or without TGF-β2 (10 ng/mL). After 6-hour stimulation, cells were collected for RNA-Seq. A total of 104 genes were upregulated by TGF-β2 in siNC-transfected cells but unchanged in siTGFBR3-transfected cells even upon TGF-β2 stimulation, which are believed to be those induced by TGF-β2 in a TGF-βR3–dependent manner. (**B**) Dot bubble graph shows the top 12 enriched GO terms based on GO analysis of 104 genes revealed in **A**. (**C**) Gene-concept network visualization of genes in the top 6 of GO-enriched terms (in black dots) and their enriched pathways (in gray lines). Red dots represent genes involved in the top 6 of GO-enriched terms and purple arrows represent collagen genes. (**D**) ALI-cultured HNECs obtained from controls were transfected with siTGFBR3 and subsequently stimulated with TGF-β2 (10 ng/mL). After 6-hour stimulation, the mRNA expression levels of *COL1A1*, *COL4A1*, *COL4A2*, and *COL5A1* were detected by RT-PCR (*n* = 6). For **D**, data are presented as the mean ± SEM and were analyzed by 1-way ANOVA with Tukey’s post hoc test. **P < 0.01.

Subsequently, we studied the downstream mRNA expression in nasal epithelial cells after activation of TGF-β2/TGF-βR3 signaling. We first compared differences between negative control siRNA-transfected (siNC-transfected) and submerged cultured HNECs with or without TGF-β2 treatment. We found that there were 118 genes upregulated by TGF-β2. We next compared the differences between siTGFBR3-transfected and submerged cultured HNECs treated with or without TGF-β2. Notably, 104 of the above 118 genes were no longer upregulated in response to TGF-β2 stimulation when the *TGFBR3* gene was knocked down, which are presumably genes induced by TGF-β2 in a TGF-βR3–dependent manner (Figure 7A). GO analysis of those 104 genes revealed that they were primarily enriched in processes such as ECM production and organization and tissue development (Figure 7B), consistent with biological processes regulated by MEX3B (Figure 2B). Further network analysis revealed that the collagen genes *COL1A1*, *COL4A1*, *COL4A2*, and *COL5A1*, were centrally located among the top 6 enriched GO terms (Figure 7C). To verify the RNA-Seq findings, we repeated the experiment using ALI-cultured HNECs and performed the RT-PCR. We confirmed that *COL1A1*, *COL4A1*, *COL4A2*, and *COL5A1* were upregulated by TGF-β2 treatment, which were abolished by siTGFBR3 treatment in ALI-cultured HNECs (Figure 7D).

### MEX3B and TGF-βR3 correlate with collagen production in eosinophilic CRSwNP.

**Figure 8:**
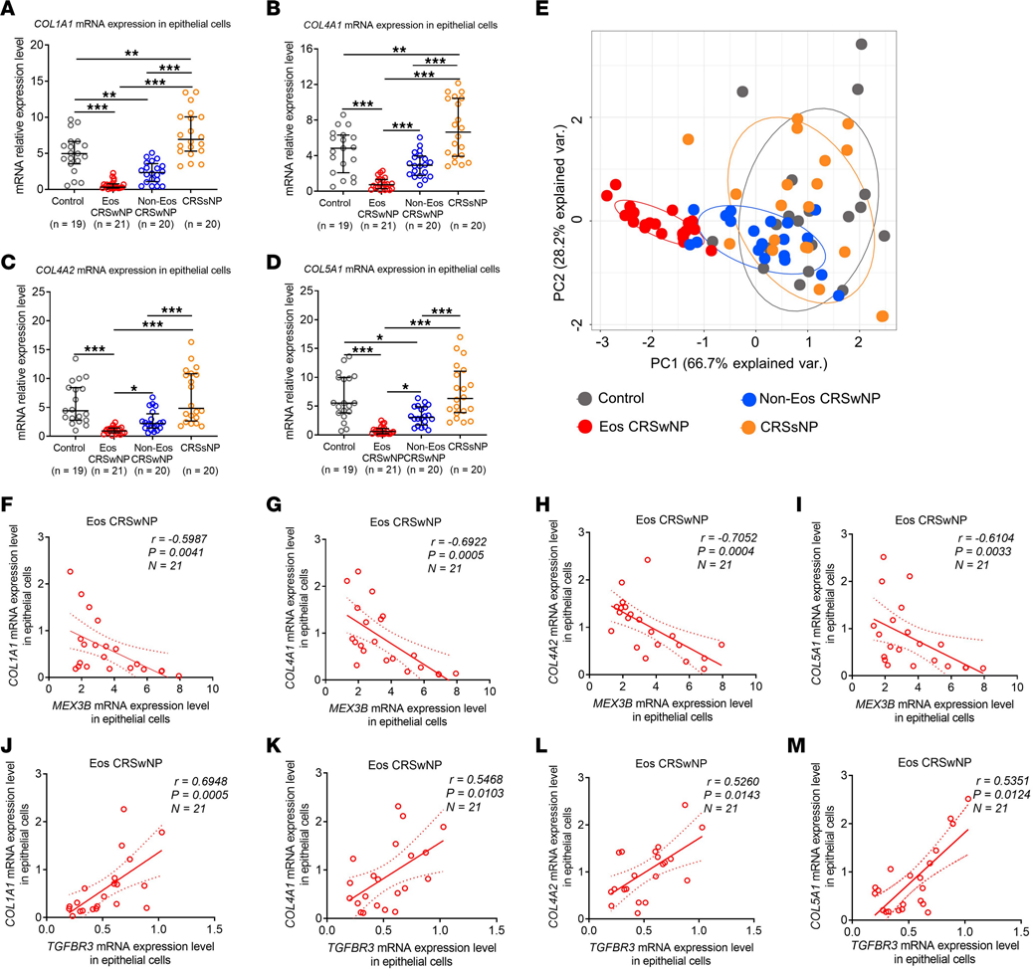
MEX3B and TGF-βR3 correlate with collagen production in eosinophilic CRSwNP. Epithelial *COL1A1* (**A**), *COL4A1* (**B**), *COL4A2* (**C**), and *COL5A1* (**D**) mRNA levels in different study groups as detected by quantitative RT-PCR. Data are presented as median and interquartile range and were analyzed by the Kruskal-Wallis test with Dunn’s post hoc test. (**E**) Principal component analysis based on the mRNA levels of *COL1A1*, *COL4A1*, *COL4A2*, and *COL5A1* in nasal epithelial cells. The *MEX3B* mRNA expression levels negatively correlated with the *COL1A1* (**F**), *COL4A1* (**G**), *COL4A2* (**H**), and *COL5A1* (**I**) mRNA expression in nasal epithelial cells in eosinophilic CRSwNP. The *TGFBR3* mRNA expression levels positively correlated with the *COL1A1* (**J**), *COL4A1* (**K**), *COL4A2* (**L**), and *COL5A1* (**M**) mRNA expression in nasal epithelial cells in eosinophilic CRSwNP. For **F**–**M**, Spearman’s correlation was used for correlation analysis. *P < 0.05, **P < 0.01, and ***P < 0.001. Eos, eosinophilic; Non-Eos, non-eosinophilic.

**Figure 9:**
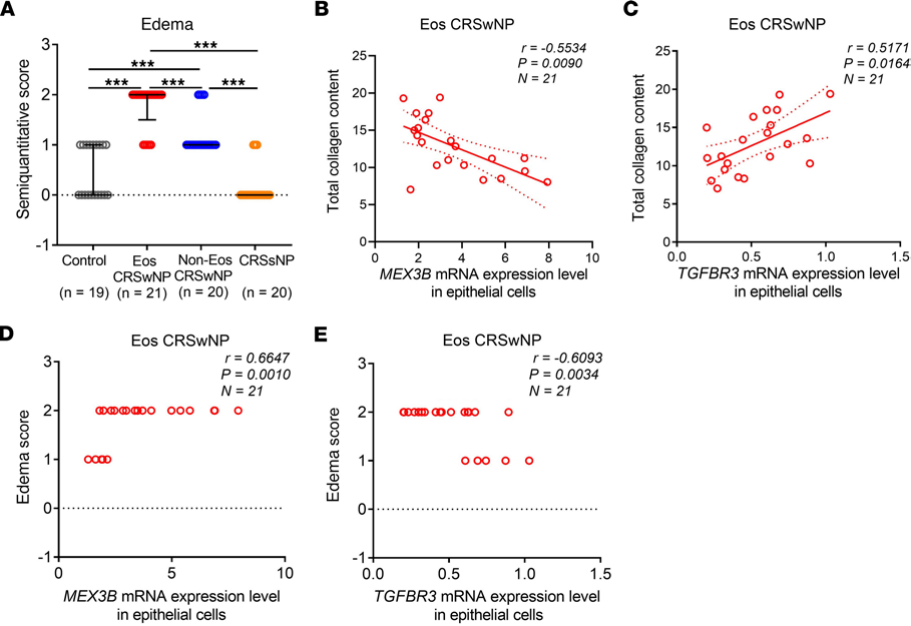
MEX3B and TGF-βR3 correlate with tissue edema severity in eosinophilic CRSwNP. (**A**) Semiquantitative evaluation of edema severity in sinonasal tissues from different study groups. Data are presented as median and interquartile range and were analyzed by the Kruskal-Wallis test with Dunn’s post hoc test. (**B**) The epithelial *MEX3B* mRNA levels negatively correlated with tissue total collagen content in eosinophilic CRSwNP. (**C**) The epithelial *TGFBR3* mRNA levels positively correlated with the total collagen content in eosinophilic CRSwNP. (**D**) The epithelial *MEX3B* mRNA levels positively correlated with tissue edema severity in eosinophilic CRSwNP. (**E**) The epithelial *TGFBR3* mRNA levels negatively correlated with the edema severity in eosinophilic CRSwNP. For **B**–**E**, Spearman’s correlation was used for correlation analysis. ***P < 0.001. Eos, eosinophilic; Non-Eos, non-eosinophilic.

Finally, we explored the expression of identified core collagen genes in different CRS subtypes. We found a significant reduction in *COL1A1*, *COL4A1, COL4A2*, and *COL5A1* gene mRNA expression in epithelial cells from patients with CRSwNP as compared with epithelial cells from controls, with a more profound reduction in eosinophilic CRSwNP than in noneosinophilic CRSwNP (Figure 8, A–D). Principal component analysis revealed clear distinctions in collagen gene expression in eosinophilic CRSwNP compared with other types of CRS and control (Figure 8E). Interestingly, we found a negative correlation between levels of mRNA for *MEX3B* and *COL1A1*, *COL4A1*, *COL4A2*, and *COL5A1*, whereas *TGFBR3* mRNA was positively correlated with these collagens in epithelial cells in patients with eosinophilic CRSwNP (Figure 8, F–M). Consistently, Picrosirius red staining showed that the total amount of collagen was markedly reduced in eosinophilic and noneosinophilic NP tissues but was increased in diseased sinus mucosa tissues from patients with CRSsNP when compared with control nasal tissues (Supplemental Figure 11). Again, a more profound decrease in collagen was observed in eosinophilic CRSwNP compared with noneosinophilic disease (Supplemental Figure 11). When we used a semiquantitative analysis of tissue edema, we discovered that the tissue edema scores were significantly higher in eosinophilic and noneosinophilic CRSwNP than in CRSsNP and controls and found higher edema in eosinophilic CRSwNP compared with noneosinophilic CRSwNP (Figure 9A). We found that epithelial *MEX3B* mRNA levels (Figure 9B) negatively correlated and epithelial *TGFBR*3 mRNA levels (Figure 9C) positively correlated with total tissue collagen amount in eosinophilic patients with CRSwNP. Consistently, we found that epithelial *MEX3B* mRNA levels were positively associated with tissue edema scores (Figure 9D), but epithelial *TGFBR*3 mRNA levels were associated negatively (Figure 9E) with edema in eosinophilic CRSwNP participants.

## Discussion

RBPs have emerged as critical players in posttranscriptional regulation of gene expression, affecting pre-mRNA splicing, mRNA turnover, and mRNA stability and translation (6–8, 10). The biology and pathological roles of MEX3B in human diseases have been poorly studied. Based on our data, we identified a potentially novel MEX3B/*TGFBR3* mRNA interaction that regulates TGF-β2 signaling and activation in human airway epithelial cells; specifically, we show that elevated MEX3B reduces TGF-βR3 and the associated TGF-β2 signaling. This disturbance of the epithelial MEX3B/*TGFBR3* pathway may contribute to the reduced collagen production and subsequent edema formation in eosinophilic CRSwNP. Our study underscores a potentially previously unrecognized role of MEX3B in the pathogenesis of CRSwNP.

Although MEX3B expression was upregulated in nasal epithelial cells in all types of CRS, the highest expression was found in eosinophilic CRSwNP. The upregulation of MEX3B expression in nasal epithelial cells in CRS may be due to the induction by CRS-relevant inflammatory cytokines or pathogens including bacteria and viruses. We found that the proinflammatory cytokine IL-1β, as well as type 2 cytokines IL-4 and IL-13, promoted MEX3B expression in nasal epithelial cells. This may account for the highest expression of MEX3B in eosinophilic CRSwNP, which is type 2 skewed. While inflammatory cytokines are increased in nearly all types of CRS, only eosinophilic CRSwNP has increased expression of type 2 cytokines in addition to proinflammatory cytokines (16, 34).

Using immunofluorescence staining and Western blotting analysis, we identified an expression of MEX3B in the cytoplasm of nasal epithelial cells, indicating that MEX3B likely exerts its effects in the cytoplasm. We found that the genes regulated by MEX3B in nasal epithelial cells are mainly involved in collagen synthesis and tissue development. By iRIP-Seq analysis, we identified *TGFBR3* mRNA as a potentially novel target of MEX3B in human nasal epithelial cells. Based on several lines of evidence, and for what we believe to be the first time, we revealed that MEX3B decreased the stability of *TGFBR3* mRNA and diminished levels of the mRNA by directly binding to AAAAAAA motifs in the 3′ UTR and destabilizing it in nasal epithelial cells. First, we demonstrated that MEX3B predominantly bound to the 3′ UTR of *TGFBR3* mRNA. Second, deletion of segments enriched with AAAAAAA motifs, or the introduction of gene point mutations into the AAAAAAA motifs in the 3′ UTR (portion 4385 to 4405 site or 5416 to 5422 site relative to the TSS) abolished the inhibitory effect of MEX3B on the activity of a luciferase reporter fused with the *TGFBR3* 3′ UTR. Third, overexpression and knockdown of MEX3B decreased or increased the stability of *TGFBR3* mRNA, respectively. The effects of MEX3B appear to be specific for *TGFBR3,* and MEX3B had no significant effect on *TGFBR1* or *TGFBR2* mRNA expression or activation of IL-13 signaling in nasal epithelial cells. In this study, we did not further determine the mechanisms underlying the influence of MEX3B on *TGFBR3* mRNA stability. Previous studies indicate that RBPs may accelerate the deadenylation and shortening of the poly-A tail, or endoribonucleolytic cleavage of mRNA, thus leading to faster degradation of mRNA (35, 36). Further investigations will be required to elucidate the mechanisms employed by MEX3B in airway epithelial cells.

The TGF-β/TGF-βR signaling pathway plays a vital role in the regulation of cell proliferation, differentiation, migration, ECM production, and cell metabolism (31, 33, 37). Aberrant expression of TGF-β/TGF-βR family members has been reported in human airway diseases including CRS (25, 30, 38); however, our current understanding of the regulation of TGF-β/TGF-βR family members is very limited, particularly for TGF-βR3. TGF-βR3 has previously been reported to be expressed on myoblasts, rat bronchiolar and alveolar epithelium, fibroblasts, macrophages, and dendritic cells (29, 39–42). Here, we demonstrate that TGF-βR3 was markedly expressed by epithelial cells rather than hematopoietic cells and stromal cells in lamina propria in sinonasal tissues. Van Bruaene et al. reported a downregulation of *TGFBR3* mRNA levels in whole NP tissues (25). Here, we specifically revealed that TGF-βR3 expression was markedly reduced in nasal epithelial cells in NPs, especially in eosinophilic type disease, and that expression was inversely correlated with levels of MEX3B.

Generally, TGF-β1 and TGF-β3 can bind with high affinity to TGF-βR2, which leads to the recruitment of TGF-βR1 and phosphorylation of SMAD transcription factors (25, 33). However, TGF-β2 has a low affinity for binding to TGF-βR2. Studies on myoblasts indicate that TGF-βR3 is a specific TGF-β2 facilitator that may promote TGF-β2 signaling by forming the TGF-β2/TGF-βR3 complex and then recruiting TGF-βR2 and TGF-βR1 (28, 29). In the current study, we also determined an indispensable role of TGF-βR3 for signaling mediated by TGF-β2, but not TGF-β1, in nasal epithelial cells. Concomitantly, we discovered that MEX3B modulated TGF-β2 signaling activation in nasal epithelial cells. Although there was an elevation of TGF-β2 levels, the TGF-β/TGF-βR signaling activation as indicated by p-SMAD2 levels was downregulated in CRSwNP, especially in the eosinophilic type. This is likely due to the downregulation of TGF-βR3 in nasal epithelial cells in eosinophilic CRSwNP, perhaps a consequence of overexpression of MEX3B.

We further determined the downstream effects of TGF-β2/TGF-βR3 activation in nasal epithetical cells. By several comparisons, we identified genes induced by TGF-β2 in a TGF-βR3–dependent way. These studies demonstrated that TGF-β2/TGF-βR3 signaling primarily regulated biological processes such as ECM production and organization in nasal epithelial cells, and expression of *COL1A1*, *COL4A1*, *COL4A2*, and *COL5A1* was a prominent effect. The collagen family comprises 28 members and can be divided into several subgroups based on their structural and functional properties (43). Type I collagen and type V collagen are fibril-forming collagens and type IV collagen is nonfibrillar and participates in basement membrane thickening in vertebrates (43). Fibrosis has been considered to be characterized by the accumulation of activated fibroblasts and excessive deposition of ECM, especially type I collagen (44). Most research has focused on mechanisms of type I collagen synthesis by fibrocytes. However, the cellular sources of type I collagen may be diverse (44). Recently, epithelial cells have been indicated as an origin of collagens in idiopathic pulmonary fibrosis (44). In the current study, we discovered that nasal epithelial cells were able to produce collagens and their importance was supported by the correlations between epithelial MEX3B and TGF-βR3 expression and the total tissue collagen amount in eosinophilic CRSwNP. Several studies indicate that type 2 cytokines (IL-4 and IL-13) are profibrotic and stimulate type 1 collagen production by fibroblasts in pulmonary fibrosis (45). In this study, we found a downregulation of collagen production in nasal epithelial cells in eosinophilic CRSwNP characterized by predominant type 2 response, suggesting distinct regulation mechanisms for collagen production in different cell types and disease contexts. We found that the expression of these collagen genes was profoundly downregulated in eosinophilic CRSwNP, which showed a clear distinct expression profile from other types of CRS and controls. The in vitro mechanisms were recapitulated in vivo by studying patient samples ex vivo. We demonstrated that the collagen production was positively correlated with TGF-βR3 levels, but inversely correlated with MEX3B levels in nasal epithelial cells collected from patients with eosinophilic CRSwNP. Notably, consistent but opposite correlations were found between tissue edema severity and epithelial MEX3B and TGF-βR3 expression in eosinophilic CRSwNP. Collectively, our results suggest that disturbance of MEX3B/TGF-βR3 in epithelial cells may lead to reduced collagen production and subsequent edema formation in CRSwNP, particularly the eosinophilic type.

MEX3B may have different functions in different cells and pathological conditions. Yamazumi et al. found that in an asthmatic mouse model with steroid-resistant neutrophilic inflammation, mice deficient for MEX3B had significantly less neutrophil infiltration and MEX3B posttranscriptionally upregulated neutrophil chemokine CXCL2 (13). Nevertheless, our data suggest an association between eosinophilic inflammation and MEX3B in CRS. We found that LPS and poly (I:C) increased the MEX3B expression in epithelial cells, indicating that bacterial and viral infection may decrease the expression of TGF-βR3 through MEX3B in CRS. In addition to MEX3B, we cannot preclude other factors or medicines that may regulate TGF-βR3 expression and TGF-β2 signaling activation in epithelial cells. Obviously, these issues require further explorations in future.

Our study has several limitations. In this study, we also observed MEX3B expression in inflammatory cells, including mast cells, macrophages, neutrophils, and B cells, in diseased sinonasal mucosa from patients with CRS. The role of MEX3B in these cells in the context of CRS remains to be explored. Although the role of epithelial MEX3B in CRSwNP would be gratifying to confirm in vivo in animal models, there is currently no adequate translational model of CRSwNP available. MEX3B is a canonical KH-domain RBP and usually binds to the 3′ UTR or 5′ UTR of targeted mRNA (46). We found that MEX3B binding sites were also enriched in the 5′ UTRs although to a lesser extent than 3′ UTRs in BEAS-2B cells. Whether MEX3B has other functions via 5′ UTRs remains to be clarified. In addition, further studies are needed to fully exclude the possibility of binding to poly-A tail of targeted mRNAs by MEX3B.

In conclusion, the present study, for what we believe to be the first time, demonstrated that MEX3B decreases epithelial *TGFBR3* mRNA expression by binding to its 3′ UTR and reducing its stability. TGF-βR3 is a TGF-β2–specific coreceptor in nasal epithelial cells, and activation of TGF-β2/TGF-βR3 signaling promotes collagen production. These results suggest a model in which overexpression of MEX3B induced by type 2 cytokines leads to TGF-βR3 downregulation and blunted TGF-β2 signaling activation in nasal epithelial cells, which reduces collagen production and promotes edema formation in eosinophilic CRSwNP. Our findings have the potential to lead to the development of therapeutic strategies targeting MEX3B, such as an antisense oligo targeting MEX3B, in the hope of improving clinical outcomes for patients with CRSwNP in the future.

## Methods

### Participants.

A total of 117 controls, 118 patients with eosinophilic CRSwNP, 113 patients with noneosinophilic CRSwNP, and 103 patients with CRSsNP were enrolled in this study. The diagnosis of CRSsNP and CRSwNP was made according to the European Position Paper on Rhinosinusitis and Nasal Polyps 2020 (14). CRSwNP was further classified as eosinophilic when the percentage of tissue eosinophils exceeded 10% of the total number of infiltrating cells, as reported by our previous study (18). Control participants were those undergoing septoplasty because of anatomic variations and did not have other sinonasal diseases. Atopic status was evaluated by using the skin prick test with a standard panel of inhalant allergens common in our region and/or using ImmunoCAP to detect IgE Abs against common inhalant allergens (Phadia) (47). The diagnosis of allergic rhinitis was based on the concordance between atopic status and typical allergic symptoms. The diagnosis of asthma was made according to the Global Initiative for Asthma guideline (48). Oral glucocorticoid and intranasal steroid sprays were discontinued at least 3 months and 1 month before surgery, respectively. Participants who had unilateral polyps such as an antrochoanal polyp, fungal sinusitis, cystic fibrosis, primary ciliary dyskinesia, immunodeficiency, or systemic vasculitis were excluded from this study. Participants with an acute upper respiratory tract infection or acute asthma episode within 4 weeks of entering the study and patients under immunotherapy were also excluded. Owing to the low prevalence of nonsteroidal antiinflammatory drug–exacerbated respiratory disease in our cohort of patients with CRS (4 out of 317), we excluded those patients in this study.

Ethmoid sinus mucosa tissues from patients with CRSsNP, NP tissues from patients with CRSwNP, and inferior turbinate mucosa tissues from controls were collected during surgery. Nasal epithelial cells were scraped from NPs of patients with CRSwNP and from the middle meatus mucosa of patients with CRSsNP and controls using a sterile Rhino-Pro curette (Arlington Scientific) as previously described (49). Each nasal scraping specimen yielded 1 × 10^6^ to 2 × 10^6^ cells, of which more than 95% were epithelial cells based on cytokeratin immunofluorescence staining with an anti–pan-cytokeratin Ab (catalog ab7753, mouse, Abcam) (50). Because of the limited quantity of tissue and cell samples, not all samples were included in every study protocol. The sample size for each experiment is indicated in the corresponding figures and figure legends. More information is provided in Supplemental Table 1.

### Histology and immunofluorescence staining.

Fresh tissue samples were fixed in formaldehyde solution and embedded in paraffin. Paraffin sections (4 μm) were prepared from tissue blocks. After deparaffinization and rehydration, sections were stained with H&E to determine the general pathologic features and to count the number of eosinophils and total inflammatory cells as previously reported (18). Edema was semiquantitatively scored on a 3-point scale, with 0 representing the lowest and 2 representing the highest scores, as mentioned elsewhere (22). Total collagens were detected by means of Picrosirius red staining, and quantified by using ImageJ software (NIH) and expressed as a percentage of the total area (25).

For immunofluorescence staining, tissue sections were stained with the primary Abs (Supplemental Table 2) and subsequent fluorescence-conjugated secondary Abs (Supplemental Table 3). Species- and subtype-matched Abs were used as negative controls. The positive staining intensity in the epithelium was analyzed by using ImageJ software, and the results are presented as the average optical density value per unit area (51). A blocking experiment with human recombinant MEX3B (1 μg/mL; Abnova) was performed to confirm the specificity of polyclonal anti-MEX3B (catalog sc-135304, rabbit, Santa Cruz Biotechnology) for immunostaining.

Immunofluorescence staining of submerged cultured HNECs was performed as previously described with minor modifications (52). Briefly, after culture, HNECs were fixed using 4% paraformaldehyde for 30 minutes and permeabilized for 20 minutes using 0.2% Triton X-100 PBS. Fixed and permeabilized cells were incubated with a primary Ab against p-SMAD2 (1:200; catalog 18338, Cell Signaling Technology) overnight at 4°C, followed by incubation with fluorophore-conjugated secondary Abs listed in Supplemental Table 3. Finally, DAPI was used to counterstain the nuclei.

Our immunofluorescence staining images were acquired at the same microscope (Olympus BX53) settings with a lack of nonlinear adjustments.

### Quantitative RT-PCR.

Total RNA was extracted from human tissue and cell samples by using TRIzol reagent (InvitroGen). A total of 1 μg of RNA was reverse-transcribed to cDNA using a PrimeScript RT reagent kit (TaKaRa Biotechnology). PCR assays were performed by using the SYBR Premix Ex Taq kit (TaKaRa Biotechnology) with appropriate primers (Supplemental Table 4). *GUSB* or *GAPDH* was used as a housekeeping gene for normalization. Relative gene expression was calculated by using the 2^–ΔΔCT^ method (53).

### Western blotting.

Total cellular protein was extracted from HNECs or BEAS-2B cells in RIPA lysis buffer containing a 2% cocktail of protease inhibitor (Guge Biotechnology). Samples containing 40 μg of proteins were separated on SDS-PAGE under reducing conditions and transferred onto PVDF membranes (Guge Biotechnology). The membranes were probed with indicated primary Abs (Supplemental Table 5) and HRP-conjugated secondary Ab (goat anti-rabbit Ab, catalog GB23303, or goat anti-mouse Ab, catalog GB23301, Guge Biotechnology) was then added. After that, the members were detected by using an ECL chemiluminescence reaction kit (Guge Biotechnology) and followed by exposure on chemiluminescent film to visualize the proteins. GAPDH was used as an internal standard to correct for variations in total protein loading. Densitometric analysis of the blots was performed by using the AlphaEase FC software (Alpha Innotech). MEX3B, TGF-βR3, SMAD2, p-SMAD2, Stat6, p-Stat6, GUSB, and GAPDH were identified as a single band at approximately 58 kDa, 92 kDa, 60 kDa, 60 kDa, 105 kDa, 105 kDa, 78 kDa, and 37 kDa on SDS-PAGE, respectively.

The cytoplasmic and nucleus proteins of nasal epithelial cells obtained from patients with eosinophilic CRSwNP were extracted separately as previously described with minor modifications (54). The HNECs were suspended in cytoplasmic extraction buffer and lysed on ice for 30 minutes. Nucleus pellets were separated from cytoplasm by centrifugation at 16,000*g* for 10 minutes. The supernatants were collected as cytoplasmic extract. After removal of cytoplasmic extracts, nucleus pellets were resuspended in the nuclei extraction buffer (containing 2% protease inhibitor cocktail) and sonicated. Then, the lysates were centrifuged at 16,000*g* for 10 minutes at 4°C, and the supernatants were collected as nucleus protein extracts.

### ELISA.

Tissue samples were weighed and 1 mL of 0.9% sodium chloride solution supplemented with 10 μL of 100 mM PMSF was added per 0.1 g tissue (5, 55). Tissue samples were homogenized on ice and centrifuged at 1,000*g* for 10 minutes at 4°C (5). Then the supernatants were harvested and stored at –80°C for future use. The TGF-β1, TGF-β2, and TGF-β3 protein levels in tissue homogenates were measured by using commercial ELISA kits (Boster Biotechnology). The lower detection limits were 15.6 pg/mL, 31.2 pg/mL, and 31.2 pg/mL for TGF-β1, TGF-β2, and TGF-β3, respectively. The total protein levels were measured by using a bicinchoninic acid protein detection kit (Guge Biotechnology), and the cytokine levels in tissue homogenates were expressed as protein levels/mg total protein.

### Cell culture, stimulation, and transfection.

An ALI culture of HNECs were obtained from controls (5, 56). HNECs obtained by nasal scrapping were washed twice in DMEM (InvitroGen) with penicillin/streptomycin and then grown submerged in bronchial epithelial cell basal medium (BEBM) (Lonza) supplemented with SingleQuot Kit Supplement Pack (Lonza) in 6-well plates coated with rat tail collagen type I (Sigma-Aldrich) in a 5% CO_2_ humidified atmosphere at 37°C. Upon confluence, the cells were passaged to collagen-coated 0.4 μm pore membrane Transwell culture plates (Becton Dickinson) and cultured with BEBM. When cells were confluent, they were shifted to ALI culture by removing all apical medium, and the medium in the basal chamber was replaced with ALI medium consisting of BEBM/DMEM H (1:1; InvitroGen) supplemented with all-trans-retinoic acid (30 ng/mL; MedChemExpress). HNECs were maintained in ALI culture for 21 days for differentiation (5, 56). After HNECs were differentiated, they were stimulated with CpG, IL-4, IL-13, IL-10, IFN-γ, TNF-α, IL-1β, and IL-17A at 10 ng/mL (R&D Systems); LPS at 1 μg/mL (InvivoGen); and poly (I:C) at 25 μg/mL (Tocris Bioscience) (5, 57). After treatment for 6 or 24 hours, the cells were harvested to detect the mRNA and protein levels of MEX3B.

For submerged cells culture, HNECs from controls were cultured submerged in BEBM in 6-well plates coated with rat tail collagen type I in a 5% CO_2_ humidified atmosphere at 37°C as previously described (58). BEAS-2B cells, a human bronchial epithelial cell line, were cultured submerged with DMEM/F-12 supplemented with 10% fetal calf serum (InvitroGen) at 37°C with 5% CO_2_ in humidified air (5). When the cells reached 80%–90% confluence, the medium was changed to serum-free medium for further study.

SiRNA transfection was performed on cells cultured submerged or cultured with the ALI method as previously described with minor modified (5, 59–61). When BEAS-2B cells or submerged cultured HNECs reached 80% confluence, the cells were transfected with siMEX3B (100 nM; Thermo Fisher Scientific), siTGFBR1 (100 nM; Viewsolid Biotech), siTGFBR2 (100 nM; Viewsolid Biotech), siTGFBR3 (100 nM; Viewsolid Biotech), or the corresponding siNC (100 nM) using Lipofectamine RNAimax (InvitroGen) for 8 hours according to the manufacturer’s instructions. At the 14th, 17th, and 21st day of differentiation, the HNECs maintained in ALI culture were pretreated with EGF (100 μg/mL; Sigma-Aldrich), a known enhancer of macropinocytosis, for 15 minutes and then were transfected with the abovementioned siRNA (siMEX3B, siTGFBR1, siTGFBR2, siTGFBR3), or the corresponding siNC using Lipofectamine 3000 (InvitroGen), for 24 hours. After a 24-hour recovery period, the medium was refreshed, and the cells were used for subsequent studies. The sense sequences were as follows: MEX3B siRNA was 5′-CCCGGAAUAAGAACACGGCACUCAA-3′, TGFBR1 siRNA was 5′-CCAUUGAUAUUGCUCCAAATT-3′, TGFBR2 siRNA was 5′-GCUUCUCCAAAGUGCAUUATT-3′, and TGFBR3 siRNA was 5′-GGUCACACUUCACCUGAAUTT-3′. After transfection with siMEX3B, cells were harvested for RNA-Seq, quantitative RT-PCR, and Western blotting assays to study the effect of MEX3B on gene expression. In other experiments, after transfection with siMEX3B, siTGFBR1, siTGFBR2, or siTGFBR3, cells were stimulated with TGF-β2 (10 ng/mL; R&D Systems) or TGF-β1 (10 ng/mL; R&D Systems) for 30 minutes and then subjected to Western blotting and flow cytometry analysis to detect the phosphorylation of SMAD2. To detect downstream genes regulated by *TGFBR3*, HNECs were treated with TGF-β2 (10 ng/mL; R&D Systems) for 6 hours after siTGFBR3 transfection and then subjected to RNA-Seq and RT-PCR.

For *MEX3B* and *GUSB* ectopic expression, the plasmid pcDNA3.1-*MEX3B* containing the human *MEX3B* gene (GenBank accession NM_032246; 2 μg) (pcMEX3B; GeneChem), pcDNA3.1-GUSB containing the human *GUSB* gene (GenBank accession NM_000181; 2 μg) (pcGUSB; GeneChem), and the control plasmid pcDNA3.1 (GeneChem) were transfected into BEAS-2B cells or ALI-cultured HNECs. The transfection reagent Lipofectamine 3000 (InvitroGen) was used for BEAS-2B cells for 48 hours according to the manufacturer’s protocol. At the 14th, 17th, and 21st day of differentiation, the HNECs maintained in ALI culture were pretreated with EGF (100 μg/mL; Sigma-Aldrich) for 15 minutes and then were transfected with pcMEX3B, pcGUSB, or the corresponding negative control pcDNA using Lipofectamine 3000 (InvitroGen) for 24 hours. Total RNA and protein were extracted after 48 hours of transfection for RNA-Seq and quantitative RT-PCR and Western blotting assays, respectively. In other experiments, cells transfected with pcMEX3B or pcGUSB were further stimulated with TGF-β2 (10 ng/mL; R&D Systems) or IL-13 (10 ng/mL; R&D Systems) for 30 minutes, and then cells were subjected to Western blotting and flow cytometry analysis to detect the phosphorylation of SMAD2 or Stat6.

### The mRNA stability assay.

The mRNA stability assay was performed on ALI-cultured HNECs as previously described with minor modifications (62). ALI-cultured HNECs transfected with siMEX3B, pcMEX3B, or their corresponding negative controls as described above. Cells were then either harvested (as time 0) without any additional treatment or further cultured in the presence of the transcriptional inhibitor actinomycin D (ActD) (5 μg/mL; AAT Bioquest) for 0.5, 1, 2, or 4 hours. Steady-state levels of *TGFBR3* mRNA were detectable by means of RT-PCR.

### Luciferase assay.

The DNA fragments from the human *TGFBR3* 3′ UTR containing the predicted MEX3B modification sites were amplified by PCR and cloned into a firefly luciferase reporter construct (pGL3 vector; Promega). Specifically, we constructed plasmid pGL3-3′ UTR (3072 to 6476 site) *TGFBR3* (pGL3-F1), pGL3-3′ UTR (3072 to 4105 site) *TGFBR3* (pGL3-F2), pGL3-3′ UTR (4106 to 5564 site) *TGFBR3* (pGL3-F3), and pGL3-3′ UTR (5565 to 6476 site) *TGFBR3* (pGL3-F4). To further investigate the specific binding sites of MEX3B, 4 mutant plasmids according to different AAAAAAA-enriched sites in the 3′ UTR of *TGFBR3* were cloned into the pGL3 vector, including pGL3-3′ UTR (4106 to 4114 site) *TGFBR3*-mutant (pGL3-MUT1), pGL3-3′ UTR (4385 to 4405 site) *TGFBR3*-mutant (pGL3-MUT2), pGL3-3′ UTR (5416 to 5422 site) *TGFBR3*-mutant (pGL3-MUT3), and pGL3-3′ UTR (5558 to 5564 site) *TGFBR3*-mutant (pGL3-MUT4).

For luciferase reporter assay, approximately 1 × 10^5^/per well HNECs from controls were seeded into 24-well plates. The above constructed plasmids (1 μg per well) were cotransfected with pcMEX3B or its negative control pcDNA (1 μg per well) by using Lipofectamine 3000 transfection agent (Thermo Fisher Scientific) according to the manufacturer’s instructions. After 48 hours, cell lysates were harvested and analyzed for luciferase activity using a dual luciferase reporter assay kit (Promega). Firefly luciferase values were normalized against Renilla luciferase activity, and the ratio of firefly/Renilla luciferase activity is presented. Each experiment was performed in triplicate and repeated 6 times.

### Flow cytometry.

Dispersed nasal tissue cells were prepared by means of mechanical dissociation (5, 58). The resulting single cell suspension was filtered and cell pellets were suspended in erythrocyte lysis buffer and incubated for 5 minutes. The cells were washed, resuspended, and stained with fixable viability stain 700 (BD Biosciences) to exclude dead cells. Then, the cells were stained with goat or mouse monoclonal Ab against human TGF-βR3, CD326, and CD45 (Supplemental Table 6) for 30 minutes at 37°C in the dark. Species- and subtype-matched Abs were used as negative controls. Epithelial cells were gated as CD326^+^CD45^–^ (5, 58, 63), hematopoietic cells were gated as CD45^+^CD326^–^ (58), stromal cells were gated as CD45^–^CD326^–^ (64, 65), and the expression of TGF-βR3 was analyzed.

ALI-cultured HNECs were transfected with siMEX3B, siTGFBR1, siTGFBR2, or siTGFBR3 and further stimulated with TGF-β2 (10 ng/mL; R&D Systems) and TGF-β1 (10 ng/mL; R&D Systems) for 30 minutes. Cells were harvested and fixed immediately by adding 1 mL of prewarmed BD Phosflow Fix Buffer I (BD Biosciences) for 15 minutes at 37°C in the dark to maintain the phosphorylation state. Cells were washed, resuspended, and stained with fixable viability stain 700 (BD Biosciences) to exclude dead cells. After that, cells were treated with 2 mL of BD Phosflow Perm Buffer I (BD Biosciences) on ice for 30 minutes in the dark and stained with PE-conjugated mouse monoclonal Ab against human p-SMAD2 (Supplemental Table 6) for 30 minutes at 37°C in the dark. The protein level of p-SMAD2 was analyzed by flow cytometry.

The stained cells were analyzed by using an LSRII flow cytometer (BD Biosciences). Fluorescence was determined for all cells in each sample after debris, dead cells, and aggregates were excluded by forward angle and side scatter gating (50). Data were analyzed using FlowJo software (TreeStar).

### RNA-Seq.

Total RNA was extracted from cultured cells by using TRIzol (InvitroGen). Library construction and sequencing were performed by Seqhealth Technology. The library products corresponding to 200–500 bps were enriched, quantified, and finally sequenced on Novaseq 6000 sequencer (Illumina) with the PE150 model.

To analyze differential expression patterns, the read count of unambiguous clean tags for each gene was calculated by using the reads per kilobase million mapped reads method (66). Gene expression was compared between cells with different treatments using the edgeR package (version 3.12.1), and genes with a corrected *P* < 0.05 and |log_2_ (fold change)| ≥ 1 between 2 groups were considered to have significantly differential expression (5). GO analysis for differentially expressed genes was implemented by KOBAS software (version: 2.1.1) with a *P* < 0.05 to judge statistically significant enrichment. All primary data have been uploaded to the NCBI’s Sequence Read Archive under the accession number PRJNA783332.

### iRIP-Seq.

IRIP-Seq was performed on BEAS-2B cells, and confirmation assay with RIP followed by RT-PCR was conducted on HNECs (67, 68). Cell lysates were cross-linked on ice with UV irradiation type C (254 nm) at 400 mJ/cm^2^. After that, the cell lysates were incubated with rabbit anti-MEX3B Ab (200 μg/mL; catalog sc-135304, Santa Cruz Biotechnology) or control rabbit IgG (catalog 3900, Cell Signaling Technology). The immunoprecipitates were incubated with protein A or G Dynabeads and the enriched MEX3B binding RNAs were extracted by using TRIzol (InvitroGen). The RNAs were used to generate a paired-end sequencing library with a ScriptSeq RNA-Seq Library Preparation Kit (Illumina) following the manufacturer’s instructions. High-throughput sequencing of the libraries was performed on an Illumina NextSeq 500 system using 150 nt paired-end sequencing by ABLife Inc.

All clean reads were then mapped to the human GRCh38 genome using TopHat (v2.1.1). Reads with overlapping regions were clustered at the genomic loci. The outputting reads were further randomly placed on the same genes to generate the maximum peak height from overlapping reads. All the observed peaks higher than those of the random max peaks (*P* < 0.01) were selected, and the target genes of MEX3B were finally determined by analyzing the location of all the MEX3B binding peaks in the human genome. A de novo motif search with the HOMER algorithm was performed. All primary data have been uploaded to the NCBI Sequence Read Archive under the accession number PRJNA783332.

### Statistics.

For continuous variables in the human tissue study, the results are presented in dot plots unless specifically stated. Symbols represent individual samples, horizontal bars represent medians, and error bars show interquartile ranges. Data distribution was tested for normality and homogeneity of variance using a Kolmogorov-Smirnov test or a Shapiro-Wilk test. Then, a Kruskal-Wallis test with a Dunn’s post hoc test for multiple comparisons and the Mann-Whitney *U* 2-tailed test for binary comparisons were employed. Spearman’s rank test was used for correlations. For dichotomous variables, a χ^2^ test or Fisher’s exact test was performed to determine the difference between groups. Cell culture data are expressed as the mean ± SEM and analyzed by 2-tailed paired or unpaired Student’s *t* test or 1-way ANOVA with the Tukey’s post hoc test. *P* < 0.05 was considered significant. Data were analyzed by SPSS 22.0 software.

### Study approval.

This study was approved by the Ethics Committee of Tongji Hospital of Huazhong University of Science and Technology, and each patient signed a written informed consent document. All work was carried out in accordance with the Declaration of Helsinki for experiments involving humans.

## Author contributions

JXL, ANC, and BL performed cell culture, IHC, Western blotting, ELISA, PCR, RIP, and RNA-Seq experiments; analyzed data; and prepared the manuscript. QY and KTS performed flow cytometry and cell culture experiments. YBL, CLG, and ZZW performed cell culture and PCR assay experiments. YY and LP performed the luciferase assay. XL, HW, MZ, and KX participated in tissue sample collection. CL and RPS participated in data discussions. NW and BL participated in data discussions and manuscript preparation. ZL designed the study and prepared the manuscript.

## Supplementary Material

Supplemental data

## Version 1 03/28/2023

In-Press Preview

## Version 2 05/08/2023

Electronic publication

## References

[1] Hewitt RJ, Lloyd CM (2021). Regulation of immune responses by the airway epithelial cell landscape. Nat Rev Immunol.

[2] Hellings PW, Steelant B (2020). Epithelial barriers in allergy and asthma. J Allergy Clin Immunol.

[3] Ordovas-Montanes J (2018). Allergic inflammatory memory in human respiratory epithelial progenitor cells. Nature.

[4] Kouzaki H (2017). Endogenous protease inhibitors in airway epithelial cells contribute to eosinophilic chronic rhinosinusitis. Am J Respir Crit Care Med.

[5] Liu JX (2020). The IL-37-Mex3B-Toll-like receptor 3 axis in epithelial cells in patients with eosinophilic chronic rhinosinusitis with nasal polyps. J Allergy Clin Immunol.

[6] Kilchert C (2020). From parts lists to functional significance-RNA-protein interactions in gene regulation. Wiley Interdiscip Rev RNA.

[7] Dominguez D (2018). Sequence, structure, and context preferences of human RNA binding proteins. Mol Cell.

[8] Castello A (2013). System-wide identification of RNA-binding proteins by interactome capture. Nat Protoc.

[9] Mugridge JS (2016). Structural basis of mRNA-cap recognition by Dcp1-Dcp2. Nat Struct Mol Biol.

[10] Conn SJ (2015). The RNA binding protein quaking regulates formation of circRNAs. Cell.

[11] Kwon SC (2013). The RNA-binding protein repertoire of embryonic stem cells. Nat Struct Mol Biol.

[12] Huang L (2018). The RNA-binding protein MEX3B mediates resistance to cancer immunotherapy by downregulating HLA-A expression. Clin Cancer Res.

[13] Yamazumi Y (2019). The RNA-binding protein Mex-3B plays critical roles in the development of steroid-resistant neutrophilic airway inflammation. Biochem Biophys Res Commun.

[14] Fokkens WJ (2020). European position paper on rhinosinusitis and nasal polyps 2020. Rhinology.

[15] Shi JB (2015). Epidemiology of chronic rhinosinusitis: results from a cross-sectional survey in seven Chinese cities. Allergy.

[16] Stevens WW (2016). Chronic rhinosinusitis with nasal polyps. J Allergy Clin Immunol Pract.

[17] Bachert C (2020). Adult chronic rhinosinusitis. Nat Rev Dis Primers.

[18] Cao PP (2009). Distinct immunopathologic characteristics of various types of chronic rhinosinusitis in adult Chinese. J Allergy Clin Immunol.

[19] Wang X (2016). Diversity of T_H_ cytokine profiles in patients with chronic rhinosinusitis: a multicenter study in Europe, Asia, and Oceania. J Allergy Clin Immunol.

[20] Bachert C (2014). ICON: chronic rhinosinusitis. World Allergy Organ J.

[21] Tomassen P (2016). Inflammatory endotypes of chronic rhinosinusitis based on cluster analysis of biomarkers. J Allergy Clin Immunol.

[22] Shi LL (2013). Features of airway remodeling in different types of Chinese chronic rhinosinusitis are associated with inflammation patterns. Allergy.

[23] Kato A (2021). Endotypes of chronic rhinosinusitis: relationships to disease phenotypes, pathogenesis, clinical findings, and treatment approaches. Allergy.

[24] Yan B (2019). Epithelium-derived cystatin SN enhances eosinophil activation and infiltration through IL-5 in patients with chronic rhinosinusitis with nasal polyps. J Allergy Clin Immunol.

[25] Van Bruaene N (2009). TGF-beta signaling and collagen deposition in chronic rhinosinusitis. J Allergy Clin Immunol.

[26] Van Bruaene N (2012). Inflammation and remodelling patterns in early stage chronic rhinosinusitis. Clin Exp Allergy.

[27] Pawankar R, Nonaka M (2007). Inflammatory mechanisms and remodeling in chronic rhinosinusitis and nasal polyps. Curr Allergy Asthma Rep.

[28] Lopez-Casillas F (1993). Betaglycan presents ligand to the TGF beta signaling receptor. Cell.

[29] Lopez-Casillas F (1994). Betaglycan can act as a dual modulator of TGF-beta access to signaling receptors: mapping of ligand binding and GAG attachment sites. J Cell Biol.

[30] Meng XM (2016). TGF-β: the master regulator of fibrosis. Nat Rev Nephrol.

[31] Derynck R, Budi EH (2019). Specificity, versatility, and control of TGF-β family signaling. Sci Signal.

[32] Sun T (2021). TGFβ2 and TGFβ3 isoforms drive fibrotic disease pathogenesis. Sci Transl Med.

[33] Hata A, Chen YG (2016). TGF-β signaling from receptors to SMADs. Cold Spring Harb Perspect Biol.

[34] Zhang N (2008). Different types of T-effector cells orchestrate mucosal inflammation in chronic sinus disease. J Allergy Clin Immunol.

[35] Du H (2016). YTHDF2 destabilizes m(6)A-containing RNA through direct recruitment of the CCR4-NOT deadenylase complex. Nat Commun.

[36] Park OH (2019). Endoribonucleolytic cleavage of m^6^A-containing RNAs by RNase P/MRP complex. Mol Cell.

[37] Batlle E, Massague J (2019). Transforming growth factor-β signaling in immunity and cancer. Immunity.

[38] Frangogiannis N (2020). Transforming growth factor-β in tissue fibrosis. J Exp Med.

[39] Liu H (2019). The t(1;10) (p22;q24) TGFBR3/MGEA5 translocation in pleomorphic hyalinizing angiectatic tumor, myxoinflammatory fibroblastic sarcoma, and hemosiderotic fibrolipomatous tumor. Arch Pathol Lab Med.

[40] Yang Z (2019). Novel role of the clustered miR-23b-3p and miR-27b-3p in enhanced expression of fibrosis-associated genes by targeting TGFBR3 in atrial fibroblasts. J Cell Mol Med.

[41] Hanks BA (2013). Type III TGF-β receptor downregulation generates an immunotolerant tumor microenvironment. J Clin Invest.

[42] Chong H (1999). Immunocytochemical localization of latent transforming growth factor-beta1 activation by stimulated macrophages. J Cell Physiol.

[43] Sorushanova A (2019). The collagen suprafamily: from biosynthesis to advanced biomaterial development. Adv Mater.

[44] Kleaveland KR (2014). Fibrocytes are not an essential source of type I collagen during lung fibrosis. J Immunol.

[45] Shao DD (2008). Pivotal advance: Th-1 cytokines inhibit, and Th-2 cytokines promote fibrocyte differentiation. J Leukoc Biol.

[46] Pereira B (2013). MEX-3 proteins: recent insights on novel post-transcriptional regulators. Trends Biochem Sci.

[47] Wang Y (2016). Allergic rhinitis control test questionnaire-driven stepwise strategy to improve allergic rhinitis control: a prospective study. Allergy.

[48] Boulet LP (2019). The Global Initiative for Asthma (GINA): 25 years later. Eur Respir J.

[49] Liao B (2015). Interaction of thymic stromal lymphopoietin, IL-33, and their receptors in epithelial cells in eosinophilic chronic rhinosinusitis with nasal polyps. Allergy.

[50] Lee HS (2009). Vascular endothelial growth factor drives autocrine epithelial cell proliferation and survival in chronic rhinosinusitis with nasal polyposis. Am J Respir Crit Care Med.

[51] Gu J (2013). Expression analysis of URI/RMP gene in endometrioid adenocarcinoma by tissue microarray immunohistochemistry. Int J Clin Exp Pathol.

[52] Jiao J (2018). Transforming growth factor-β1 decreases epithelial tight junction integrity in chronic rhinosinusitis with nasal polyps. J Allergy Clin Immunol.

[53] Wang ZZ (2020). Stromal cells and B cells orchestrate ectopic lymphoid tissue formation in nasal polyps. Allergy.

[54] Chiou YS (2016). Directly interact with Keap1 and LPS is involved in the anti-inflammatory mechanisms of (-)-epicatechin-3-gallate in LPS-induced macrophages and endotoxemia. Free Radic Biol Med.

[55] Cao PP (2014). Increased local IgE production induced by common aeroallergens and phenotypic alteration of mast cells in Chinese eosinophilic, but not non-eosinophilic, chronic rhinosinusitis with nasal polyps. Clin Exp Allergy.

[56] Chustz RT (2011). Regulation and function of the IL-1 family cytokine IL-1F9 in human bronchial epithelial cells. Am J Respir Cell Mol Biol.

[57] Liu Z (2009). Clara cell 10-kDa protein expression in chronic rhinosinusitis and its cytokine-driven regulation in sinonasal mucosa. Allergy.

[58] Wang H (2021). Defective STING expression potentiates IL-13 signaling in epithelial cells in eosinophilic chronic rhinosinusitis with nasal polyps. J Allergy Clin Immunol.

[59] Ota K (2015). Synthetic double-stranded RNA induces interleukin-32 in bronchial epithelial cells. Exp Lung Res.

[60] Krishnamurthy S (2012). Manipulation of cell physiology enables gene silencing in well-differentiated airway epithelia. Mol Ther Nucleic Acids.

[61] Bartman CM (2021). Passive siRNA transfection method for gene knockdown in air-liquid interface airway epithelial cell cultures. Am J Physiol Lung Cell Mol Physiol.

[62] Weidensdorfer D (2009). Control of c-myc mRNA stability by IGF2BP1-associated cytoplasmic RNPs. RNA.

[63] Mulligan JK (2018). C3a receptor antagonism as a novel therapeutic target for chronic rhinosinusitis. Mucosal Immunol.

[64] Wang ZZ (2020). B cell-activating factor promotes B cell survival in ectopic lymphoid tissues in masal polyps. Front Immunol.

[65] Cheng HW (2018). CCL19-producing fibroblastic stromal cells restrain lung carcinoma growth by promoting local antitumor T-cell responses. J Allergy Clin Immunol.

[66] Mortazavi A (2008). Mapping and quantifying mammalian transcriptomes by RNA-Seq. Nat Methods.

[67] Wang X (2021). The m6A reader IGF2BP2 regulates macrophage phenotypic activation and inflammatory diseases by stabilizing TSC1 and PPAR γ. Adv Sci (Weinh).

[68] Li W (2017). The FOXN3-NEAT1-SIN3A repressor complex promotes progression of hormonally responsive breast cancer. J Clin Invest.

