# Supplementary material for: MEX3B inhibits collagen production in eosinophilic nasal polyps by downregulating epithelial cell TGFBR3 mRNA stability
Source: JCI Insight. 2023 May 8;8(9):e159058. doi: 10.1172/jci.insight.159058 (PMC10243817; doi:10.1172/jci.insight.159058)
Supplement: Supplemental data [file jciinsight-8-159058-s113.pdf]

Supporting Information

MEX3B inhibits collagen production in eosinophilic nasal polyps by downregulating epithelial cell *TGFBR3* mRNA stability

Jin-Xin Liu [1*], Ao-Nan Chen [1*], Qihong Yu [2,3], Ke-Tai Shi [1], Yi-Bo Liu [1], Cui-Lian Guo [1], Zhe-Zheng Wang [1], Yin Yao [1], Li Pan [1], Xiang Lu [1], Kai Xu [1], Heng Wang [1], Ming Zeng [1], Chaohong Liu [4,1], Robert P. Schleimer [5,6], Ning Wu [7,8,1#], Bo Liao [1#], Zheng Liu [1#]

[1]Department of Otolaryngology-Head and Neck Surgery, Tongji Hospital, Tongji Medical College, Huazhong University of Science and Technology, Wuhan, China

[2]Hepatic Surgery Center, Tongji Hospital, Tongji Medical College, Huazhong University of Science and Technology, Wuhan, China

[3]Clinical Medical Research Center of Hepatic Surgery at Hubei Province, Wuhan, China

[4]Department of Pathogen Biology, School of Basic Medicine, Tongji Medical College, Huazhong University of Science and Technology, Wuhan, China

[5]Division of Allergy-Immunology, Department of Medicine, Northwestern University Feinberg School of Medicine, Chicago, IL, USA

[6]Department of Otolaryngology, Northwestern University Feinberg School of Medicine, Chicago, IL, USA

[7]Department of Immunology, School of Basic Medicine, Tongji Medical College,

Huazhong University of Science and Technology, Wuhan, China
[8]Cell Architecture Research Center, Huazhong University of Science and Technology, Wuhan, China

* These authors contributed equally to the completion of this article.
[#] Co-corresponding authors

For correspondence, please contact:
Zheng Liu, M.D., Ph.D., Department of Otolaryngology-Head and Neck Surgery, Tongji Hospital, Tongji Medical College, Huazhong University of Science and Technology, No. 1095 Jiefang Avenue, Wuhan 430030, China, Tel: 86-27-83662606, Fax: 86-27-83662606, E-mail: zhengliuent@hotmail.com
And
Bo Liao, M.D., Ph.D., Department of Otolaryngology-Head and Neck Surgery, Tongji Hospital, Tongji Medical College, Huazhong University of Science and Technology, No. 1095 Jiefang Avenue, Wuhan 430030, China, Tel: 86-27-83663807, E-mail: emsliaobo@163.com
And
Ning Wu, Ph.D., Department of Immunology, School of Basic Medicine, Tongji Medical College, Huazhong University of Science and Technology, Wuhan, China, Tel: 86-27-83692611, E-mail: wuning118@gmail.com

This PDF file includes:

Figs. S1 to S11

Table S1 to S6

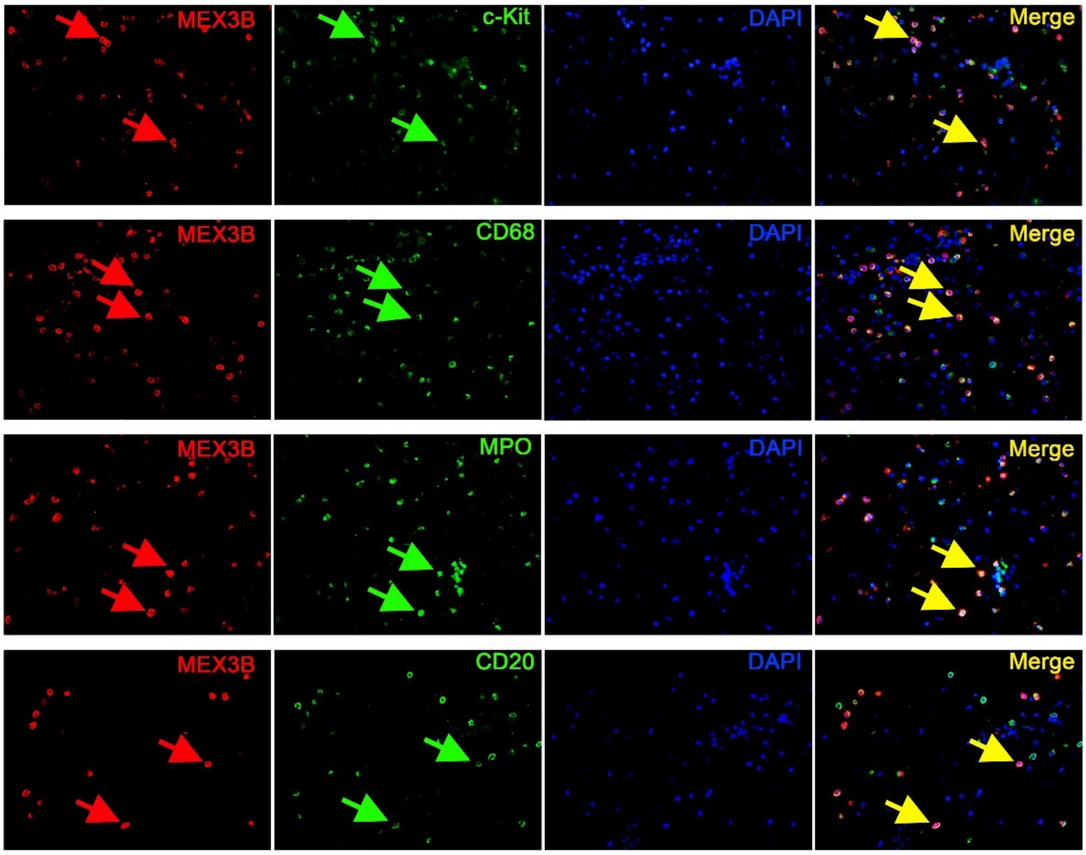

Fig. S1. MEX3B positive cells in lamina propria in sinonasal mucosa tissues. Double immunofluorescence staining demonstrated that MEX3B is expressed by c-Kit positive mast cells, CD68 positive macrophages, myeloperoxidase (MPO) positive neutrophils, and CD20 positive B cells. Representative photomicrographs show immunostaining of tissue sections from patients with eosinophilic CRSwNP (original magnification × 400). Arrows indicate the representative double positive cells.

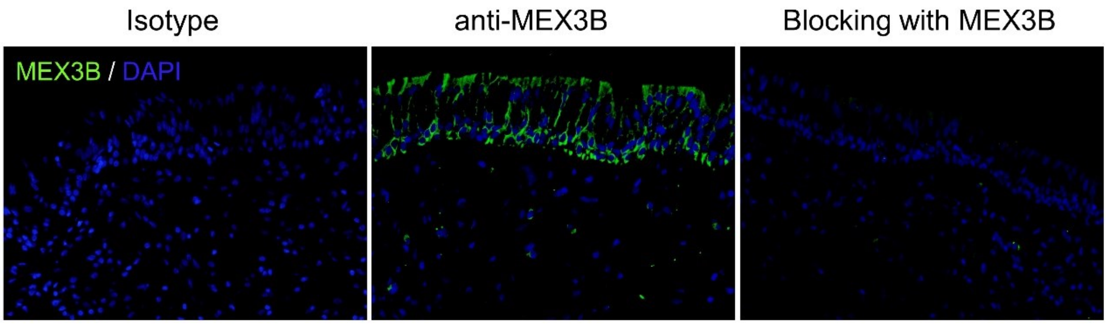

Fig. S2. Blocking experiment with human recombinant MEX3B protein to confirm the specificity of polyclonal anti-MEX3B for immunostaining. Representative photomicrographs of eosinophilic NP samples are shown (original magnification × 400).

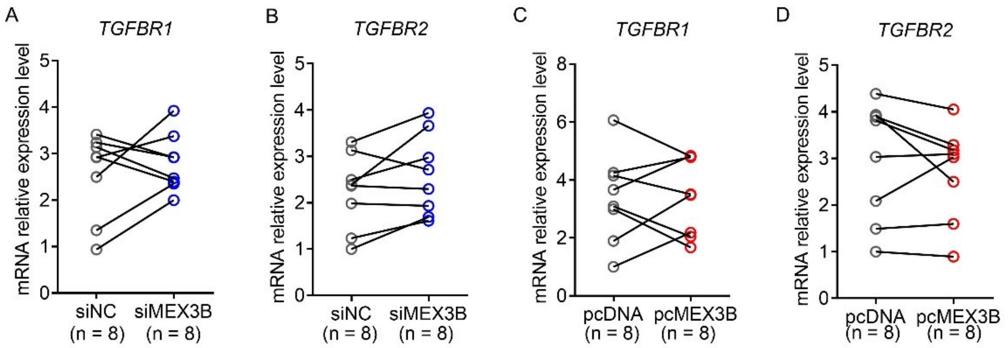

Fig. S3. MEX3B has no significant effect on *TGFBR1* and *TGFBR2* mRNA expression in HNECs. A-B, After siMEX3B transfection, *TGFBR1* (A) and *TGFBR2* (B) mRNA levels in ALI cultured HNECs were detected by RT-PCR (n = 8). **C-D,** After pcMEX3B transfection, *TGFBR1* (C) and *TGFBR2* (D) mRNA levels in ALI cultured ALI cultured HNECs were detected by RT-PCR (n = 8). For **A-D**, data are presented in dot plots and were analyzed by paired Student's t test. Symbols represent individual samples.

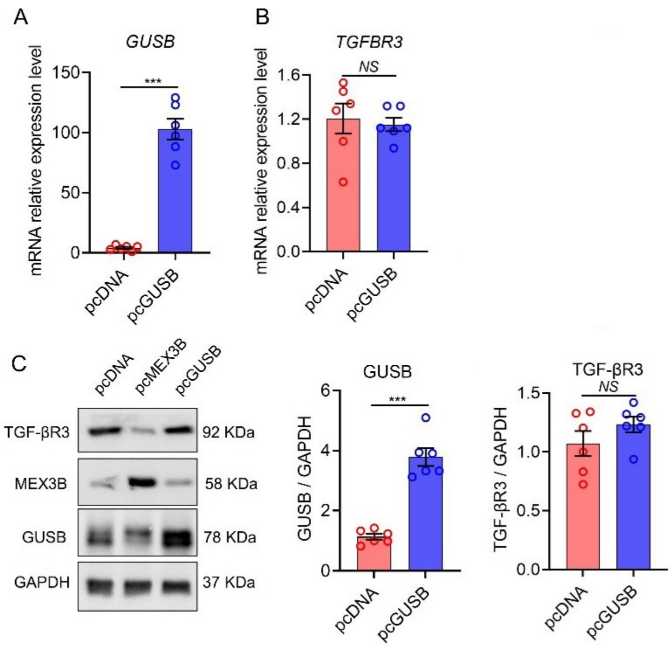

Fig. S4. pcGUSB has no effect on the expression of *TGFBR3* in ALI cultured HNECs from control subjects. A, pcGUSB transfection efficiency in HNECs was confirmed at mRNA level by RT-PCR (n = 6). **B,** After pcGUSB transfection, *TGFBR3* mRNA expression in HNECs was detected by RT-PCR (n = 6). **C,** After pcGUSB transfection, TGF-βR3 protein expression in HNECs was detected by western blotting (n = 6). Representative blots are shown and densitometric analysis of blots was performed. GUSB: beta-glucuronidase. Data are presented as the mean and SEM, and were analyzed by the unpaired Student's t test. ***P < 0.001; NS, not significant.

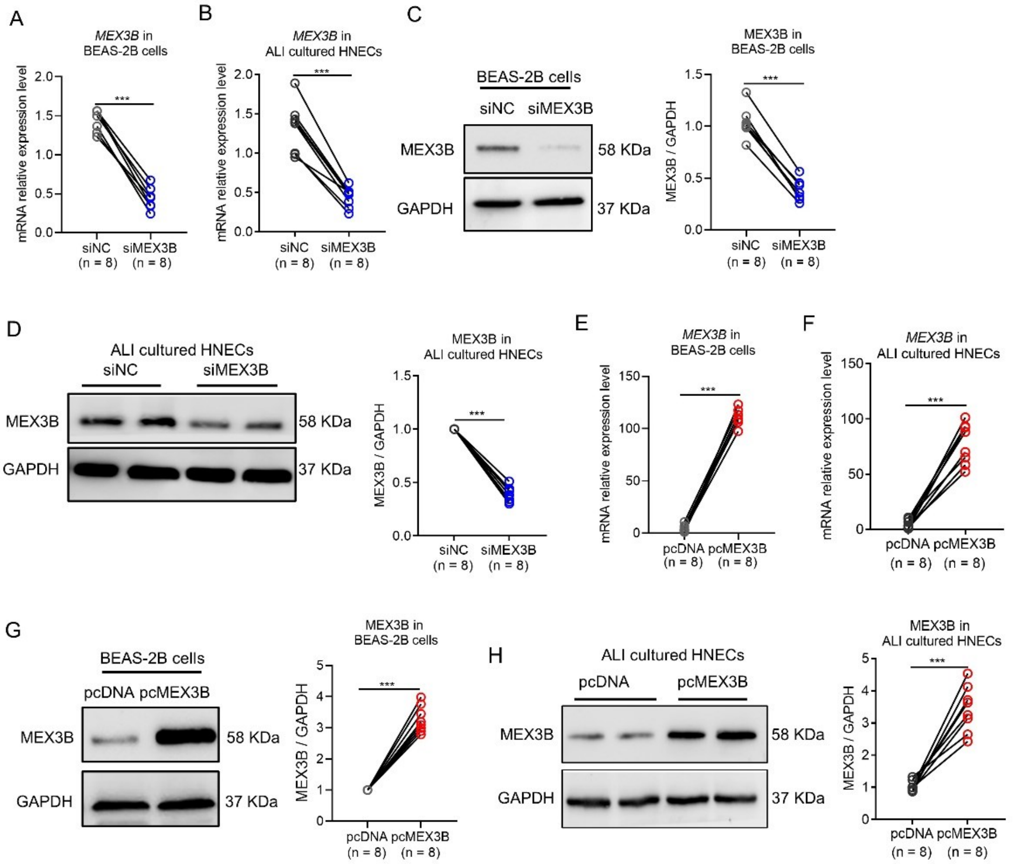

Fig. S5. The transfection efficiency of siMEX3B and pcMEX3B in ALI cultured **HNECs and BEAS-2B cells**. **A and B,** siMEX3B transfection efficiency in BEAS-2B cells (**A**) or ALI cultured HNECs (**B**) was confirmed at mRNA levels by RT-PCR, respectively (n = 8). **C and D**, siMEX3B transfection efficiency in BEAS-2B cells (**C**) or ALI cultured HNECs (**D**) was confirmed at protein levels by western blotting, respectively (n = 8). Representative blots are shown and densitometric analysis were performed. **E and F,** pcMEX3B transfection efficiency in BEAS-2B cells (**E**) or ALI cultured HNECs **(F)** was confirmed at mRNA levels by RT-PCR, respectively (n = 8). **G and H**, pcMEX3B transfection efficiency in BEAS-2B cells (**G**) or ALI cultured HNECs (**H**) was confirmed at protein levels by western blotting, respectively (n = 8). Representative blots are shown and densitometric analysis were performed. Data are presented as in dot plots and were analyzed by paired Student's t test. Symbols represent individual samples. ***$P < 0.001$.

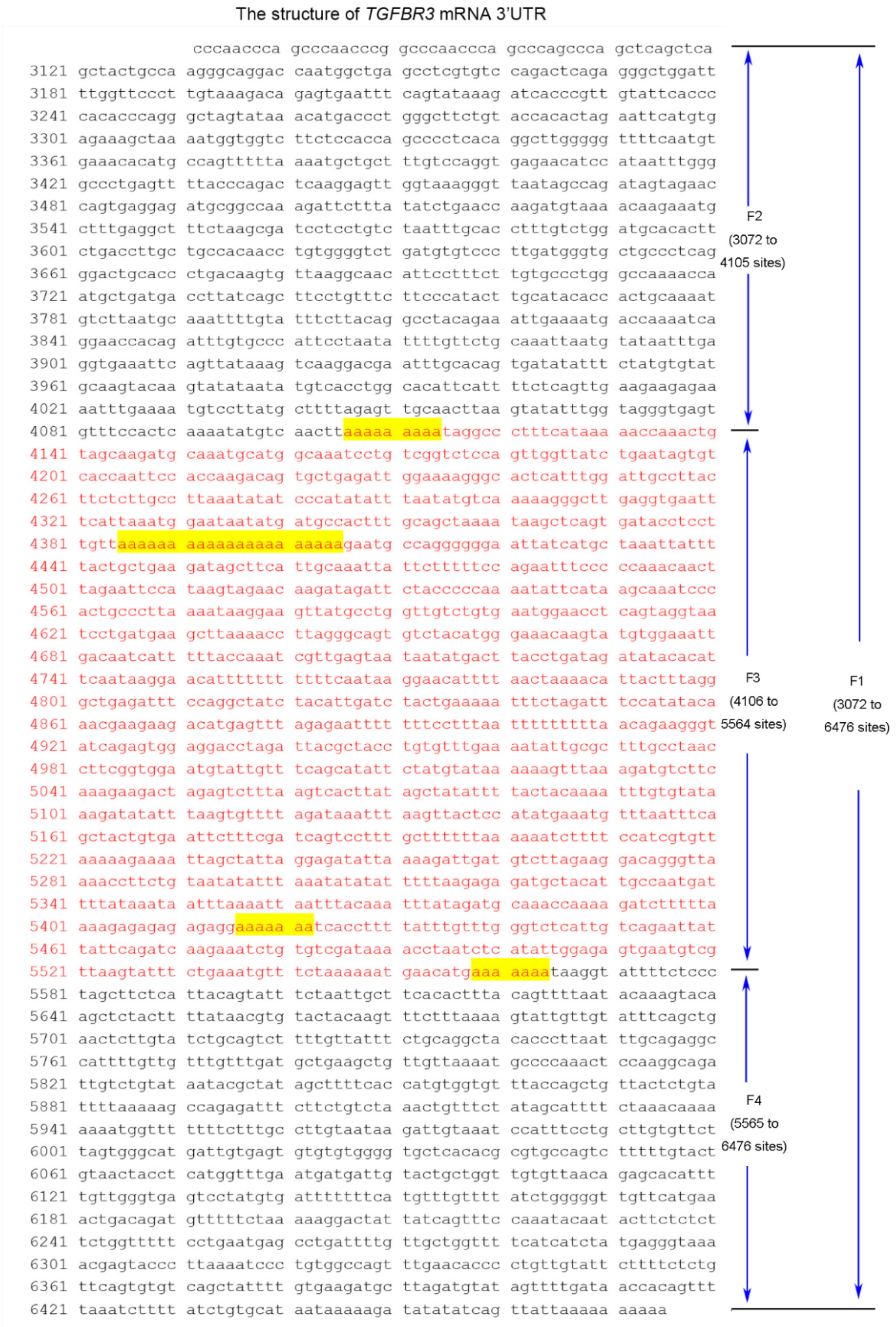

Fig. S6. The information about the 3'UTR of *TGFBR3* mRNA. The full length of 3'UTR of *TGFBR3* mRNA was denoted as the F1 segment. The 3072 to 6476 site, 4106 to 5564 site, and 5565 to 6476 site in *TGFBR3* 3'UTR were denoted as the F2, F3, and F4 segment, respectively. The putative MEX3B binding sites enriched with AAAAAAA motif were mainly concentrated in the F3 segment and highlighted with yellow color.

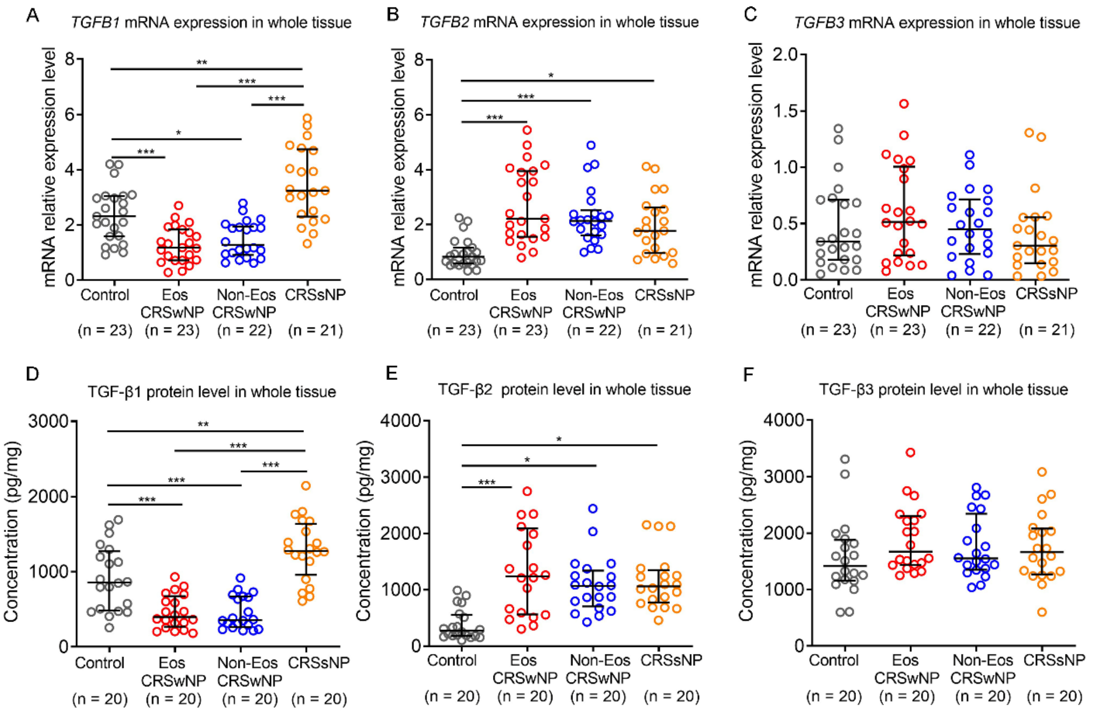

Fig. S7. The mRNA and protein expression of *TGFB1, 2 and 3* in sinonasal tissues in different study groups. A-C, *TGFB1* (A)*, TGFB2* (B)*, and TGFB3* (C) mRNA expression in sinonasal tissues from different study groups as detected by quantitative RT-PCR. **D-F,** The protein levels of TGF-β1 (D), TGF-β2 (E) and TGF-β3 (F) in sinonasal tissue homogenates from different study groups as measured by ELISA. Data are presented as median and interquartile range, and were analyzed by Kruskal-Wallis test with the Dunn *post hoc* test. *P < 0.05, **P < 0.01 and ***P < 0.001. Eos, eosinophilic; Non-Eos, non-eosinophilic.

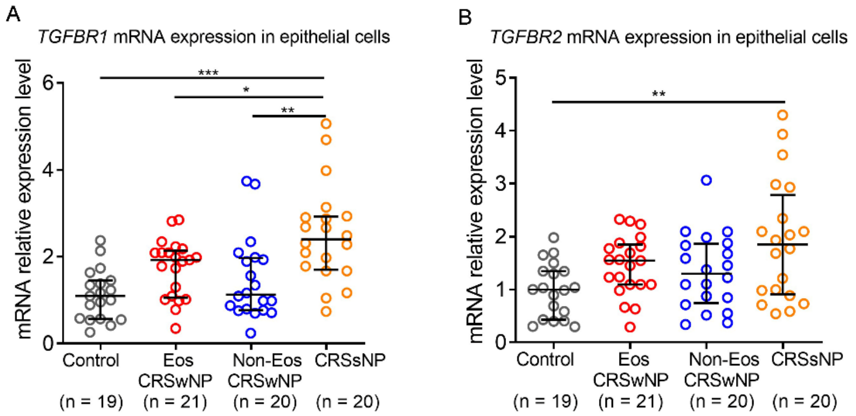

Fig. S8. The expression of *TGFBR1* and *TGFBR2* mRNA in nasal epithelial cells in different study groups. - Data are presented as median and interquartile range, and were analyzed by Kruskal-Wallis test with the Dunn *post hoc* test. $^*P < 0.05$, $^{**}P < 0.01$, and $^{***}P < 0.001$. Eos, eosinophilic; Non-Eos, non-eosinophilic.

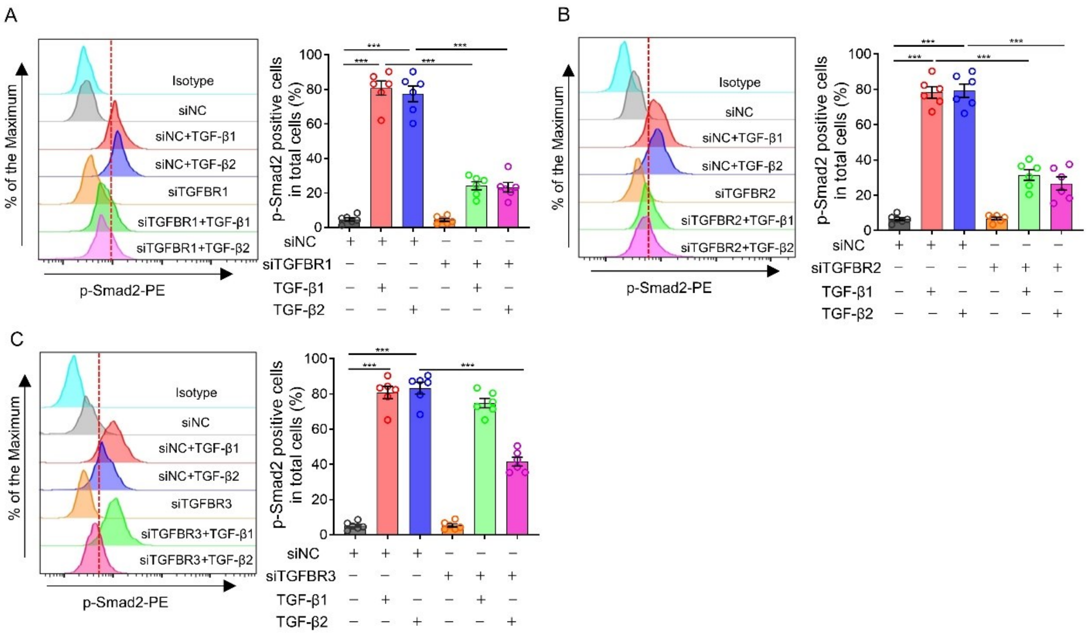

Fig. S9. The p-Smad2 protein levels in HNECs detected by flow cytometric analysis. ALI cultured HNECs were transfected with siTGFBR1, siTGFBR2 or siTGFBR3 and stimulated with TGF-β1 (10 ng/mL) or TGF-β2 (10 ng/mL). After 30-minute simulation, the p-Smad2 levels were detected by flow cytometric analysis (n = 6). Representative histograms are shown. Data are presented as the mean and SEM, and were analyzed by one-way ANOVA with the Tukey's *post hoc* test. ***$P < 0.001$.

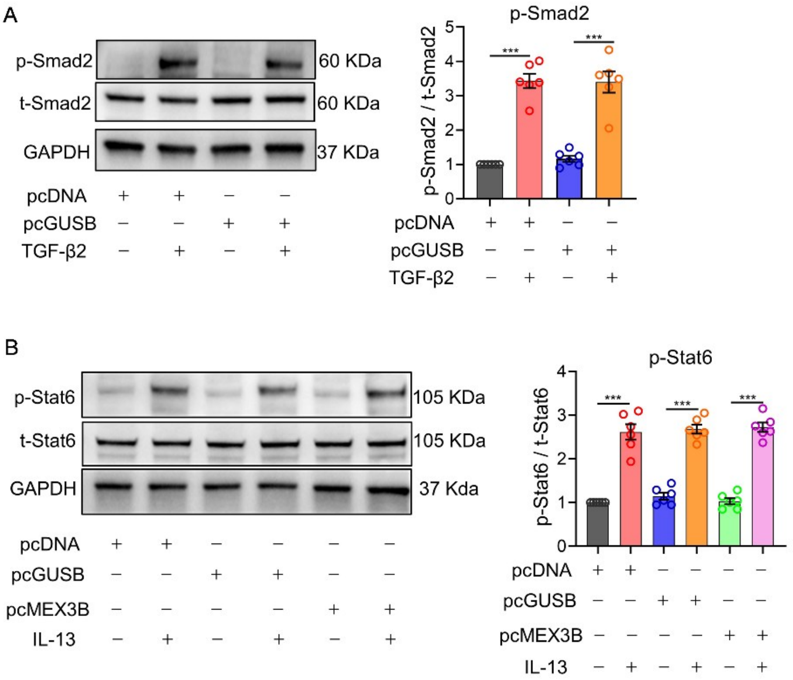

Fig. S10. The effect of pcGUSB or pcMEX3B on Smad2 or Stat6 phosphorylation. **A,** ALI cultured HNECs obtained from control subjects were transfected with pcGUSB, and then stimulated with TGF-β2 (10 ng/mL). Thirty minutes after simulation, the p-Smad2 level was detected by western blotting (n = 6). Representative blots are shown and densitometric analysis was performed. **B,** ALI cultured HNECs obtained from control subjects were transfected with pcGUSB or pc MEX3B, and then stimulated with IL-13 (10 ng/mL). Thirty minutes after simulation, the p-Stat6 level was detected by western blotting (n = 6). Representative blots are shown and densitometric analysis was performed. *GUSB*: beta-glucuronidase. Data are presented as the mean and SEM, and were analyzed by one-way ANOVA with the Tukey's post hoc test. ****P* < 0.001.

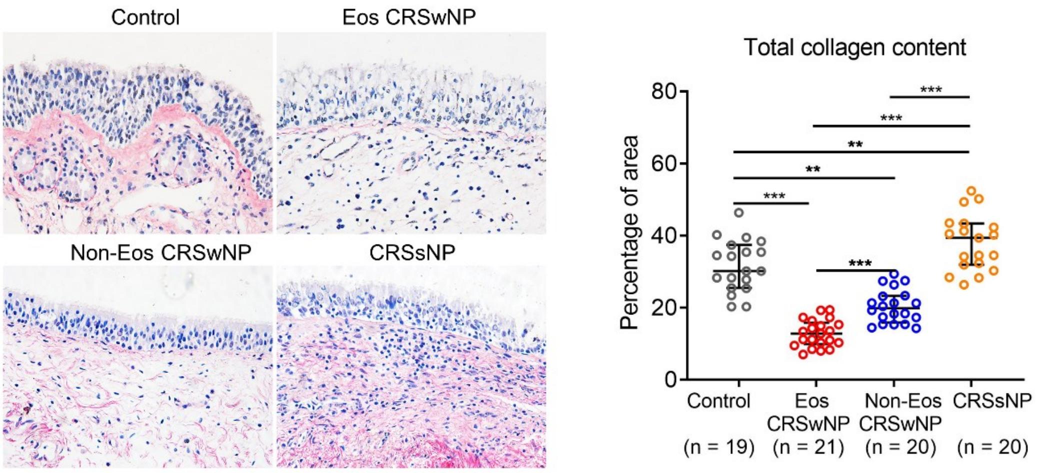

Fig. S11. The total collagen amount in sinonasal mucosa tissues in different study groups. Picrosirius red staining was performed to measure total collagen deposition, and quantified by means of ImageJ software. The representative photomicrographs are shown (original magnification × 400). Data are presented as median and interquartile range, and were analyzed by Kruskal-Wallis test with the Dunn *post hoc* test. **P < 0.01 and ***P < 0.001. Eos, eosinophilic; Non-Eos, non-eosinophilic.

Table S1. Demographic characteristics of subjects involved in different experiments

	Control	Eos CRSwNP	Non-Eos CRSwNP	CRSsNP	*P* value
**Total subjects enrolled**	117	118	113	103	
**Methodology used**					
Histology, immunohistochemistry and immunofluorescence					
Subject number	26	34	31	30	
Gender, male	23 (88%)	24 (71%)	15 (48%)	17 (57%)	*0.056*
Age (years)	37 (31-45)	43 (29-51)	40 (30-50)	39(27-48)	*0.401*
Patients with atopy	2 (7.7%)	11 (32%)	6 (19%)	7 (23%)	*0.141*
Patients with AR	0 (0)	5 (14.7%)	1 (3.2%)	4 (13%)	*0.100*
Patients with asthma	0 (0)	8 (23.5%)	1 (3.2%)	1 (3%)	***0.002***
RT-PCR					
Subject number	33	38	33	30	
Gender, male	25 (75.8%)	28 (73.7%)	22 (66.7%)	17 (56.7%)	*0.350*
Age (years)	31 (26-38)	39 (32-48)	45 (24-53)	35(25-46)	*0.05*
Patients with atopy	7 (21.2%)	11 (28.9%)	10 (30.3%)	10 (33.3%)	*0.738*
Patients with AR	0 (0)	5 (13.2%)	3(9.1%)	3 (10%)	*0.226*
Patients with asthma	0 (0)	4 (10.5%)	0 (0)	1 (3.3%)	*0.059*
Western blotting					
Subject number	20	23	21	20	
Gender, male	17 (85%)	18 (78.3%)	14 (66.7%)	12 (60%)	*0.274*
Age (years)	28 (21-37)	41 (30-50)	41 (19-47)	41(32-53)	***0.037***
Patients with atopy	6 (30%)	11 (47.8%)	7 (33.3%)	5 (25%)	*0.424*
Patients with AR	0 (0)	6 (26.1%)	3 (14.3%)	5 (25%)	*0.088*

Patients with asthma	0 (0)	3 (13%)	0 (0)	1 (5%)	*0.137*
ELISA					
Subject number	20	20	20	20	
Gender, male	16 (80%)	14 (70%)	11 (55%)	12 (60%)	*0.348*
Age (years)	30 (26-36)	43 (29-54)	41 (23-47)	37(21-43)	*0.071*
Patients with atopy	0 (0)	0 (0)	0 (0)	2 (10%)	*0.104*
Patients with AR	0 (0)	3 (15%)	0 (0)	2 (10%)	*0.124*
Patients with asthma	0 (0)	4 (20%)	1 (5%)	1 (5%)	*0.090*
Picrosirius red staining					
Subject number	10	10	10	10	
Gender, male	8 (80%)	7 (70%)	7 (70%)	5 (50%)	*0.642*
Age (years)	26 (22, 31)	41 (33, 48)	31 (24, 39)	34 (28, 46)	*0.135*
Patients with atopy	2 (20%)	2 (20%)	4 (40%)	3 (30%)	*0.865*
Patients with AR	0 (0)	1 (10%)	1 (10%)	2 (20%)	*0.891*
Patients with asthma	0 (0)	1 (10%)	0 (0)	1 (10%)	*1.000*
Flow cytometry					
Subject number	10	12	11	10	
Gender, male	8 (80%)	9(75%)	7 (63.6%)	4(40%)	*0.249*
Age (years)	26 (24-34)	45 (38-53)	50 (43-60)	42(38-49)	***0.021***
Patients with atopy	2 (20%)	4 (33.3%)	3 (27.3%)	4 (40%)	*0.841*
Patients with AR	0 (0)	2 (16.7%)	1 (8.3%)	1 (10%)	*0.893*
Patients with asthma	0 (0)	2 (16.7%)	0 (0)	1 (10%)	*0.541*
Cell culture study					
Subject number	28		-	-	**-**
Gender, male	17 (60.7%)		-	-	**-**
Age (years)	27 (23-34)		-	-	**-**
Patients with atopy	4 (14.3%)		-	-	**-**
Patients with AR	0 (0)		-	-	**-**
Patients with asthma	0 (0)		-	-	**-**

For continuous variables, results are expressed as medians and interquartile ranges. Categorical variables are summarized using percentage. Eos, eosinophilic; Non-Eos, non-eosinophilic.

Table S2. Primary antibodies used in immunofluorescence staining

Antibody	Species	Concentration	Clone ID	Reference	Source
MEX3B	rabbit	1:50	polyclonal	sc-135304	Santa Cruz Biotechnology (Santa Cruz, CA, USA)
MEX3B	mouse	1:50	D-12	sc-515833	Santa Cruz Biotechnology
TGF-βR3	mouse	1:50	MM0057-5G9	ab78421	Abcam (Cambridge, UK)
c-Kit	mouse	1:100	C117/370	ab187371	Abcam
CD68	mouse	Undiluted	KP1	ZM-0060	Zhongshan Golden Bridge Biotechnology (Beijing, China)
MPO	mouse	1:100	2C7	Ab25989	Abcam
CD20	mouse	1:100	L26	M-0039	Zhongshan Golden Bridge Biotechnology
p-Smad2	rabbit	1:100	4087	Q15796	Cell Signaling Technology (Danvers, MA, USA)

MEX3B: Mex3 RNA binding family member B; TGF-β: transforming growth factor β; TGF-βR3: TGF-β receptor III; p-Smad2: phosphorylation Smad2; IF, immunofluorescence.

Table S3. Secondary antibodies used in immunofluorescence staining

Antibody	Concentration	Clone ID	Reference	Source
IFKine™ Green donkey anti-rabbit IgG	1:100	polyclonal	A24221	Abbkine Scientific Company (Wuhan, China)
IFKine™ Red donkey anti-mouse IgG	1:100	polyclonal	A24411	Abbkine
IFKine™ Red donkey anti-rabbit IgG	1:100	polyclonal	A24421	Abbkine

Table S4. Primers used in quantitative PCR analysis

Primer	Primer sequences	Expected product size (bp)	Annealing temperature (°C)
MEX3B	(F)5'-AAGAGCGTGAACATGACCGAG -3'	90	60
	(R)5'-CGCTTTGATTTTACAACCTTGCC-3'		
TGFBR3	(F)5'-CCTTCCGTTTCCTTTCCCAGA-3'	170	60
	(R)5'-CACATTTGACAGACAGGGCAAT-3'		
TGFBR3	(F)5'- CTCAAGGAGTTGGTAAAGGGTT-3'	176	60
(3'UTR)	(R)5'- TGGCAGCAAGGTCAGAAGTG-3		
TGFBR3	(F)5'- ACTTTCCTCTTCCCAGCGAGTG-3'	133	59
(5'UTR)	(R)5'- CGGCAAAACTACGCCATCC-3'		
TGFBR3	(F)5'- CTTCCTGTTTCTTCCCATAC-3'	197	60
(CDS)	(R)5'- GCAAATTCGTCCTTGACT-3'		
TGFBR1	(F)5'-GCTGTATTGCAGACTTAGGACTG-3'	90	60
	(R)5'-TTTTTGTTCCCACTCTGTGGTT-3'		
TGFBR2	(F)5'- GCAGGTGGGAACTGCAAGAT-3'	132	60
	(R)5'- AAGGACTCAACATTCTCCAAATTC-3'		
TGFB1	(F)5'- CTAATGGTGGAAACCCACAACG-3'	209	60
	(R)5'- TATCGCCAGGAATTGTTGCTG-3'		
TGFB2	(F)5'- CAGCACACTCGATATGGACCA-3'	113	60
	(R)5'- CCTCGGGCTCAGGATAGTCT-3'		
TGFB3	(F)5'- ACTTGCACCACCTTGGACTTC-3'	114	60
	(R)5'- GGTCATCACCGTTGGCTCA-3'		
GUSB	(F)5'-ACCCAGAAGACTGTGGATGG-3'	201	60
	(R)5'-TTCTAGACGGCAGGTCAGGT-3'		
GAPDH	(F)5'-AGGTCGGTGTGAACGGATTTG-3'	95	62
	(R)5'-GGGGTCGTTGATGGCAACA-3'		
COL1A1	(F)5'- GAGGGCCAAGACGAAGACATC -3'	140	62
	(R)5'- CAGATCACGTCATCGCACAAC -3'		
COL4A1	(F)5'- GGACTACCTGGAACAAAAGGG -3'	240	60

	(R)5’- GCCAAGTATCTCACCTGGATCA -3’		
COL4A2	(F)5’- TTATGCACTGCCTAAAGAGGAGC-3’	207	60
	(R)5’- CCCTTAACTCCGTAGAAACCAAG-3’		
COL5A1	(F)5’- GCCCGGATGTCGCTTACAG-3’	80	60
	(R)5’- AAATGCAGACGCAGGGTACAG-3’		

MEX3B: Mex3 RNA binding family member B; *TGFB*: transforming growth factor β ; *TGFBR*: TGF-β receptor; *GUSB*: beta-glucuronidase; *GAPDH*: glyceraldehyde 3-phosphate dehydrogenase; *COL1A1*: collagen, type I, alpha 1; *COL4A1*: collagen, type IV, alpha 1; *COL4A2*: collagen, type IV, alpha 2; *COL5A1*: collagen, type V, alpha 1.

Table S5. Primary antibodies used in western blotting

Antibody	Species	Concentration	Clone ID	Reference	Source
MEX3B	rabbit	1:500	polyclonal	sc-135304	Santa Cruz Biotechnology (Santa Cruz, CA, USA)
MEX3B	mouse	1:500	D-12	sc-515833	Santa Cruz Biotechnology
TGF-βR3	mouse	1:500	MM0057-5G9	ab78421	Abcam (Cambridge, UK)
p-Smad2	rabbit	1:1000	18338	E8F3R	Cell Signaling Technology (Danvers, MA, USA)
Smad2	rabbit	1:1000	5339	D43B4	Cell Signaling Technology
GAPDH	mouse	1:1000	P04406	BM1623	Boster Biotechnology (Wuhan, China)
Histone H3	rabbit	1:1000	polyclonal	GB11026	Guge Biotechnology, (Wuhan, China)
p-Stat6	rabbit	1:1000	9361	Try641	Cell Signaling Technology
Stat6	rabbit	1:1000	5397	D3H4	Cell Signaling Technology
GUSB	rabbit	1:1000	EPR10616	ab166904	Abcam

MEX3B: Mex3 RNA binding family member B; TGF-β: transforming growth factor β; TGF-βR3: TGF-β receptor III; p-Smad2: phosphorylation Smad2; GAPDH: Glyceraldehyde 3-phosphate dehydrogenase; p-Stat6: phosphorylation Stat6; *GUSB*: beta-glucuronidase.

Table S6. Primary antibodies used in flow cytometry

Antigen-Fluorophore	Manufacturer	Clone ID	Source	Isotype	Dilution
TGF-βR3-PE	R&D systems, (Minneapolis, MN, USA)	FAB242P	goat	IgG	1:20
p-Smad2-PE	BD Biosciences (Franklin Lakes, NJ, USA)	O72-670	mouse	IgG1, κ	1:20
CD326-APC	Biolegend (San Diego, CA, USA)	9C4	mouse	IgG2b, κ	1:20
CD45-PerCP-Cy5.5	BD Biosciences	HI30	mouse	IgG1, κ	1:20

TGF-βR3: transforming growth factor-beta receptor III; p-Smad2: phosphorylation Smad2.
